# Isolation and Identification of Pigment-Producing Endophytic Fungi from the Amazonian Species *Fridericia chica*

**DOI:** 10.3390/jof10010077

**Published:** 2024-01-19

**Authors:** Dorothy Ívila de Melo Pereira, Raiana Silveira Gurgel, Anne Terezinha Fernandes de Souza, Rosiane Rodrigues Matias, Lucas de Souza Falcão, Francisco Celio Maia Chaves, Gilvan Ferreira da Silva, José Gregorio Martínez, Rudi Emerson de Lima Procópio, Cleiton Fantin, Patrícia Melchionna Albuquerque

**Affiliations:** 1 Programa de Pós-Graduação em Biodiversidade e Biotecnologia, Escola Superior de Ciências da Saúde, Universidade do Estado do Amazonas, Manaus 69050-010, Brazil; dorothyivila@gmail.com (D.Í.d.M.P.); raianagurgel@hotmail.com (R.S.G.); 2 Grupo de Pesquisa Química Aplicada à Tecnologia, Escola Superior de Tecnologia, Universidade do Estado do Amazonas, Manaus 69050-020, Brazil; anne.fernandes13@gmail.com (A.T.F.d.S.); rosiane.matias12@gmail.com (R.R.M.); lucas.sfalcao@hotmail.com (L.d.S.F.); rprocopio@uea.edu.br (R.E.d.L.P.); 3 Programa de Pós-Graduação em Biotecnologia e Recursos Naturais da Amazônia, Escola Superior de Ciências da Saúde, Universidade do Estado do Amazonas, Manaus 69050-010, Brazil; cfantin@uea.edu.br; 4 Embrapa Amazônia Ocidental, Manaus 69010-970, Brazil; celio.chaves@embrapa.br (F.C.M.C.); gilvan.silva@embrapa.br (G.F.d.S.); 5 Grupo de Investigación Biociencias, Institución Universitaria Colegio Mayor de Antioquia, Medellin 050001, Colombia; jose.martinez@colmayor.edu.co; 6 Programa Multicêntrico de Pós-Graduação em Bioquímica e Biologia Molecular, Escola Superior de Ciências da Saúde, Universidade do Estado do Amazonas, Manaus 69050-010, Brazil

**Keywords:** endophytes, natural colorants, fungal metabolites, phylogenetic analysis

## Abstract

Pigments of fungal origin have aroused increasing interest in the food dye and cosmetic industries since the global demand for natural dyes has grown. Endophytic microorganisms are a source of bioactive compounds, and Amazonian plant species can harbor fungi with a wide range of biotechnological applications. Popularly known in Brazil as crajiru, *Fridericia chica* is a medicinal plant that produces a red pigment. In this study, a total of 121 fungi were isolated in potato dextrose agar from three plants. We identified nine pigment-producing endophytic fungi isolated from branches and leaves of *F. chica.* The isolates that showed pigment production in solid media were molecularly identified via multilocus analysis as *Aspergillus welwitschiae*, *A. sydowii*, *Curvularia* sp., *Diaporthe cerradensis* (two strains), *Hypoxylon investiens*, *Neoscytalidium* sp. (two strains) and *Penicillium rubens.* These isolates were subjected to submerged fermentation in two culture media to obtain metabolic extracts. The extracts obtained were analyzed in terms of their absorbance between 400 and 700 nm. The pigmented extract produced by *H. investiens* in medium containing yeast extract showed maximum absorbance in the red absorption range (UA_700_ = 0.550) and significant antioxidant and antimicrobial activity. This isolate can thus be considered a new source of extracellular pigment.

## 1. Introduction

Plants harbor interrelated microorganisms, such as bacteria and fungi, which form plant microbiomes [1]. Among these microorganisms are endophytic fungi. These are commonly found in the different tissues of plants and interact in such a way as to not cause damage to the hosts [2,3]. In this symbiotic association between fungi and plants, the latter provide carbohydrates to endophytic fungi, which in turn perform beneficial functions for the hosts, such as the production of secondary metabolites that protect the plant against diseases, herbivores, and biotic and abiotic stresses, in addition to promoting host growth [4,5,6]. On the other hand, although endophytic fungi behave mutually, they can occasionally become pathogenic, depending on the environmental and physiological conditions of the host plant [2,7].

Endophytic fungi have aroused increasing interest in the biotechnological and industrial fields due to their ability to secrete secondary metabolites with interesting properties. Their metabolites can be used as biocontrol agents, antimicrobials, antitumors, antioxidants, antidiabetic agents, antibiotics, and insecticides, among others [5,8]. Therefore, endophytic fungi represent important sources for the discovery of new natural products with bioactive properties [9].

Among the fungal metabolites of interest, pigments have attracted attention from the dye industries due to their antimicrobial and antioxidant properties, and because they have low or no toxicity [10,11]. In addition, there is a tendency to replace synthetic dyes with natural ones since the negative side effects caused by synthetic dyes have already been proven [12,13].

Filamentous endophytic fungi are capable of producing a wide variety of biocolors, such as melanins, phenazins, flavins, carotenoids, quinones, violacein, indigo, monascins, rubropuntamine, rubropuntatin and ankaflavin [13,14]. In addition, fungi exhibit relatively rapid growth and release pigments into the culture medium, which makes it possible to obtain metabolic extracts and produce them on an industrial scale. Differently to extracting pigments from plants or animals, this process is more economical and does not depend on climatic conditions or seasonality [10].

The genus *Fridericia* is distributed from Mexico and the Antilles to northern Argentina [15] and is commonly found in the Amazon rainforest and Atlantic Forest of Brazil. The species *Fridericia chica* (Bonpl.) L. G. Lohmann, popularly known in Brazil as crajiru, pariri, or carajuru, among other names, is a plant that is widely used due to its tinctorial, medicinal and cosmetic properties. *F. chica* was first described by Cronquist [16] as *Arrabidaea chica*. However, its classification has been modified due to recent taxonomic changes [17]. 

Three different varieties of *F. chica* are cultivated in the Amazon region [18]. Its medicinal properties are described in several studies [19,20,21,22]. Due to its pharmacological properties, *F. chica* was included by the Brazilian Ministry of Health in the National List of Medicinal Plants of Interest to the Unified Health System (RENISUS), along with 71 others that are recognized as being medicinal plants. 

In a recent study conducted by our research group, we identified the antimicrobial and antioxidant potential of metabolites produced by endophytic fungi of *F. chica* [8]. Despite this, there are still knowledge gaps in relation to the identification and evaluation of the capacity for pigment production in these endophytic fungi. It is known that endophytic fungi associated with *F. chica* have the potential for pigment production, since the host plant is a producer of 3-deoxyanthocyanins, such as carajurine (6,7-dihydroxy-5,4′-dimethoxyflavylium) and carajurone (6,7,4′-trihydroxy-5-methoxyflavylium), which are red pigments used as chemical markers of the species [17,18]. 

Therefore, in order to contribute to new discoveries related to fungi associated with this plant host as potential sources of dyes, in this study, we describe the isolation, selection and identification of extracellular pigment-producing endophytic fungi associated with *F. chica*. 

## 2. Materials and Methods

### 2.1. Collection of Plant Material

Variety II of *Fridericia chica* (Bignoniaceae) was used as the host plant. Healthy aerial parts (fresh leaves and branches) were collected from three bushes in a plantation of Embrapa Western Amazon, located on the AM-010 highway, Km 29 (Manaus–Itacoatiara highway), following the procedure described by Araújo et al. [23]. The location of the three bushes was determined using geographical coordinates obtained through the Global Positioning System (GPS): shrub 1 (02.88930° S/059.96759° W), shrub 2 (02.88927° S/059.96759° W) and shrub 3 (02.88927° S/059.96751° W). The collection was carried out in February 2019. 

### 2.2. Isolation of Endophytic Fungi

The isolation was registered for scientific research in the National System for the Management of Genetic Heritage and Associated Traditional Knowledge (SISGEN), under the code A0B4857. The isolation of *F. chica* endophytic fungi followed the procedure described by Araújo et al. [23]. Initially, the plant material, composed of leaves and branches, underwent a superficial disinfection process. This process consisted of washing the material under running water with a neutral detergent. Then, fragments of 10 to 12 cm were subjected to a sequence of immersions in different solutions, in the following order and period: for the leaves, 70% alcohol for 1 min, 3% sodium hypochlorite for 2.5 min, again 70% alcohol for 30 s and, finally, sterile distilled water for 2 min; for the branches, 70% alcohol for 1 min, 4% sodium hypochlorite for 3 min, 70% alcohol for 30 s and, finally, sterile distilled water for 2 min. 

After the superficial disinfection, the plant material was cut into small fragments of approximately 6 mm^2^ and inoculated onto Petri dishes containing sterile PDA culture medium. Six fragments were placed on each plate. The plates containing the fragments were kept in an incubator (NT 705, Novatécnica, Piracicaba, Brazil) at a temperature of 26 °C for 15 days. As a test of the surface sterilization, aliquots of water (50 µL) from the last washing of the plant fragments were sown on PDA medium. These samples also were incubated at a temperature of 26 °C for 15 days.

During the incubation period, the fungi that showed growth were isolated in Petri dishes containing PDA. Then, the fragments were maintained for another 15 days at 26 °C to allow the development and macroscopic observation of the colonies of fungi with distinct characteristics. These colonies were purified using the striae depletion technique in order to obtain isolated colonies.

The 121 endophytic fungi (78 from leaves and 43 from branches) isolated from *F. chica* were deposited in the Central Microbiological Collection of the Amazonas State University (CCM/UEA), where they were preserved using the Castellani method [24].

### 2.3. Selection of Pigment-Producing Fungi in a Solid Medium

For the selection of endophytic fungi that produce extracellular pigment in a solid medium, the *F. chica* isolates were initially reactivated in Petri dishes containing PDA and incubated at 26 °C for 2 to 5 days. The pure isolates were transferred to new Petri dishes containing PDA and incubated at 26 °C for 14 days. After the growth period, the fungi were evaluated via macroscopic observation for the production of extracellular pigment diffused in the culture medium, following the method described by Heo et al. [25]. The presence of pigments in the mycelium was also checked. All the isolates that showed pigment production in a solid medium were selected for the evaluation of pigment production in submerged fermentation.

### 2.4. Pigment Production in Submerged Fermentation

The pigment-producing isolates selected via the solid medium screening were inoculated from three fungal culture fragments (5 × 5 mm in diameter) in 100 mL Erlenmeyer flasks containing 50 mL of liquid medium. In order to evaluate the most suitable nitrogen source for the production of fungal pigments, two liquid media were used, both with the pH adjusted to 5.0. The YEX medium consisted of white potato (200 g/L), dextrose (10 g/L) and yeast extract (2.0 g/L), as described by Bose, Gowrie and Chathurdevi [26], with modifications. The PEP medium was composed of white potato (200 g/L), dextrose (10 g/L) and peptone (2.0 g/L) [27]. The cultures were maintained under static conditions at 30 °C for 14 days. 

### 2.5. Extraction of Pigments from the Culture Medium

After cultivation in liquid medium, 20 mL of chloroform and 20 mL of ethyl acetate were added sequentially to each culture broth containing the fungal mycelium. The cultures containing solvents were shaken at 100 rpm for 24 h in the dark. After this time, the phases were separated using a separation funnel. The pigmented extracts were then filtered with a Millipore membrane with a pore size of 0.45 µm and subjected to an absorbance reading between 400 and 700 nm in a UV/VIS spectrophotometer (UV 1800, Shimadzu, Kyoto, Japan) in order to determine the maximum absorbance of the pigment produced (λmax) [28].

### 2.6. Evaluation of the Biological Activities of the Promising Pigmented Extract

The most promising pigmented extract was evaluated in terms of its antioxidant and antimicrobial properties. For the evaluation of biological activities, 30 fungal mycelium fragments (5 × 5 mm in diameter), which were removed from the PDA plates, were inoculated into 1000 mL Erlenmeyer flasks containing 500 mL of YEX medium [26], pH 4.0. The cultures were maintained under static conditions at 25 °C for 18 days. After cultivation, the extraction was carried out as described in Section 2.4. After the solvent was rotaevaporated, the extract was resuspended at 10 mg/mL with a 10% dimethyl sulfoxide (DMSO) solution and stored at −18 °C for later use in the biological tests.

For the evaluation of antioxidant activity, two different methods were used: the 2,2-diphenyl-1-picrylhydrazyl radical sequestration (DPPH•) method and the ferric reducing antioxidant power (FRAP) method. In the DPPH• test, the solution of this radical was prepared at a concentration of 0.06 mmol/L using methanol P.A. and was protected from direct exposure to light [29]. The procedure was conducted in 2 mL Eppendorf microtubes, in which 200 µL of the extract was added, followed by the addition of 1250 µL of the DPPH• solution. For the negative control, 200 µL of 10% DMSO and 1250 µL of DPPH• solution were added [30]. The microtubes were shielded from direct light and, after 15 min, the absorbance readings were recorded on a UV/VIS spectrophotometer (UV 1800, Shimadzu, Kyoto, Japan) at 517 nm. These experiments were conducted in triplicate. The pigmented fungal extract was initially tested at a single concentration of 10 mg/mL. Quercetin served as the standard and was used at a concentration of 40 µg/mL. The percentage of DPPH• radical sequestration was calculated using Equation (1), using the values of the drop in the absorbance of the sample (*Abs_sample_*) and the control (*Abs_control_*). The effective concentration for the sequestration of 50% of DPPH• radicals (EC_50_) was determined, which was calculated from successive dilutions of the sample and by generating a linear regression graph. Quercetin was tested in a concentration range of 100 to 3125 µg/mL.

(1) $$AA\;\left(\%\right)=\frac{{Abs}_{control}-{Abs}_{sample}}{{Abs}_{control}}\times 100$$

For the analysis of the ferric reducing antioxidant power, the method described by Benzie and Strain was used, with modifications [31]. An aliquot of 2.45 mL of the FRAP reagent was incubated with 0.35 mL of the sample for 30 min, at 37 °C, under protection from light. Subsequently, the samples were evaluated using a UV-VIS spectrophotometer (UV 1800, Shimadzu, Kyoto, Japan) at 595 nm. The FRAP reagent was composed of 100 mL of acetate buffer (0.3 mM), 10 mL of TPTZ (10 mM) and 10 mL of aqueous ferric chloride solution (20 mM). All the experiments were conducted in triplicate. The pigmented extract was tested at a single concentration of 10 mg/mL, and a standard curve was constructed using Trolox in 10% DMSO. The results were expressed in µmol of Trolox equivalent per gram of extract (µmol TE/g). As a standard, ascorbic acid was used at a concentration of 40 µg/mL, and 10% DMSO was used as a blank.

To evaluate the antimicrobial activity, the microdilution method was used, according to the guidelines of the Clinical and Laboratory Standards Institute (CLSI) [32], which included the reduction of resazurin for antibacterial assays and the reduction of TTC for antifungal assays. Commercially obtained strains, such as *Escherichia coli* CCCD-E005, *Serratia marcescens* CCCD-S005, *Proteus mirabilis* CCCD-P001, *Pseudomonas aeruginosa* CCCD-P004, *Bacillus subtilis* CCCD-B005, *Staphylococcus aureus* CCCD-S009, *Klebsiella pneumoniae* CCCD-K003, *Salmonella enterica* CCCD-S003, *Candida albicans* CCCD-CC001 and *C. tropicalis* CCCD-CC002, were tested.

The procedure was performed in a 96-well microplate containing 100 µL of the ex-tract in different concentrations (10, 5.0, 2.5, 1.25, 0.625 and 0.312 mg/mL) and 100 µL of microbial inoculum. The microbial inoculum was prepared from colonies cultured for 24 h, with the suspension standardized at 0.5 on the McFarland scale (10^8^ CFU/mL) and diluted in the culture medium (Mueller–Hinton broth for bacteria and Sabouraud broth for fungi) until reaching 5 × 10^5^ CFU/mL. Levofloxacin at 0.25 mg/mL was used as a positive control for bacteria, and terbinafine at 0.40 mg/mL for fungi. The negative control consisted of only the microbial inoculum and, for the sterility control, 100 µL of the sterile culture medium used in the preparation of the inoculum was added to the wells. The solvent used to dilute the extract was 10% DMSO [8].

Following this, the plates were incubated at 37 °C for 24 h (bacteria) and 48 h (fungi) in a BOD chamber. After the addition of 30 µL of 0.01% resazurin or 1% 2,3,5-triphenyltetrazolium chloride (TTC), the plates were incubated again at 37 °C for 1–2 h to observe the color change resulting from the reduction of the coloring reagents. The minimum inhibitory concentration (MIC) was determined using successive dilutions of the sample. The lowest concentration of the extract that inhibited microbial growth was considered the MIC.

### 2.7. Chemical Profile of the Active Pigmented Extract

The active pigmented extract was analyzed via thin layer chromatography (TLC) to identify the main chemical classes present in the fungal extract. Then, 50 mg of the pigmented extract was dissolved in 500 µL of methanol. Using capillaries, 2 µL of the samples was placed on a silica gel chromatographic plate (TLC aluminum sheets, Macherey-Nagel, 20 × 20 cm, silica gel 60 matrix, fluorescent indicator). Dichloromethane:methanol (95:5) was used as the mobile phase.

To detect the chemical classes, ultraviolet light at 254 nm and 365 nm and the following chemical developers were used: ferric chloride, aluminum chloride, ceric sulfate, and vanillin/H_2_SO_4_. Ferric chloride was obtained by diluting 1.5 g of FeCl_3_ in 50 mL of ethyl alcohol. Aluminum chloride was prepared with 1 g of AlCl_3_ in 100 mL of ethyl alcohol. To prepare the ceric sulfate, 2.1 g of cerium IV sulfate was dissolved in 25 mL of distilled water, and 1.4 mL of concentrated H_2_SO_4_ was added, followed by heating. After cooling, this solution was adjusted to 50 mL with distilled water. To prepare the vanillin/H_2_SO_4_, 5 mL of sulfuric acid was mixed with 100 mL of ethanol (solution I) and 1 g of vanillin was dissolved in 100 mL of ethanol (solution II). The plate was then sprayed with solution I and then with solution II under heating [33,34].

Fourier-transform infrared spectrophotometry (FTIR) was performed in order to verify the functional groups of the pigmented extract. A wavelength range of 400 to 4000 cm^−1^, with resolution of 1 cm^−1^ and 70 scans, was used in a spectrophotometer (IRAffinity-1S, Shimadzu, Kyoto, Japan). The extract was analyzed using a KBr pellet, compressed by a 15-ton hydraulic press (CrushIR, Pike Technologies, Fitchburg, WI, USA).

### 2.8. Molecular Identification of Pigment-Producing Endophytic Fungi from Fridericia chica

The fungi selected as pigment producers in the screening in a solid medium were identified via sequencing the internal transcribed spacer regions (ITS), β-tubulin (*tub*2), calmodulin (*cal*), partial gene of elongation factor 1-alpha (*tef*1) and second largest protein subunit of DNA-directed RNA polymerase II (*rpb*2). The total DNA was extracted using the CTAB method [35], with modifications. 

Following the protocol described by Oetari et al. [36], with modifications, the DNA amplification via PCR (polymerase chain reaction) had a final reaction volume of 15 µL. Here, 3 mM MgCl_2_, 0.2 mM dNTPs, 1× buffer 10×, 0.2 µM forward primer, 0.2 µM reverse primer, 1 U Taq polymerase and 50 ng of fungal genomic DNA were used. The annealing temperatures and sequences of each of the primers (*Its*1 and *Its*4, Bt-2a and Bt-2b, CALM-228F and CALM-737R, EF1-728F and EF1-986R, RPB2-6F and fRPB2-7cR) used in this study are described in Table 1. 

The amplification conditions consisted of the following steps: an initial denaturation cycle of 5 min at 95 °C; followed by 35 cycles in three steps: (1) denaturation at 95 °C for 30 s, (2) annealing at 54 °C, 58 °C or 62 °C, depending on the specific hybridization temperature for each primer, for 30 s, followed by (3) extension at 72 °C for 1 min. Finally, a cycle was included with a final extension step at 72 °C for 5 min. 

The amplified PCR product was purified with 20% PEG 8000 and the sequences were read with an automatic sequencer (ABI 3130xl Genetic Analyzer, Applied Biosystems, Thermo Fisher, Waltham, MA, USA). The sequences were manually checked, aligned, edited and analyzed with the help of Bioedit 7.2.6 [41]. As reference standards, sequences obtained from the BLASTn database deposited in the GenBank at the NCBI were used (www.ncbi.nlm.nih.gov, (accessed on on 24 August 2023)) in order to obtain a preliminary identification and sequences with high identity to the type specimens, which were representative for phylogenetic reference. The newly obtained sequences were deposited in GenBank.

### 2.9. Phylogenetic Analysis

For the construction of the phylogenetic tree, the sequences were aligned using the MAFFT program (version 7) (https://mafft.cbrc.jp/alignment/software/ (accessed on 24 August 2023)) [42]. The concatenation of the loci (ITS + *tub*2 + *cal* + *tef*1 + *rpb*2) was carried out with the aid of the program MEGA (version X) [43]. Once the multilocus database was obtained, phylogenetic reconstruction was performed using the IQ-Tree program (http://iqtree.cibiv.univie.ac.at/, (accessed on 24 August 2023)) [44] via maximum likelihood (ML) analysis. The ML analysis included 1000 replicates (bootstrap) using all the sites, based on the molecular evolution model previously estimated via the ModelFinder program [45]. The resulting trees were visualized using FigTree v. 1.4.4 (http://tree.bio.ed.ac.uk/software/figtree/ (accessed on 25 September 2023)) and edited using the Inkscape program (vector graphics editor).

## 3. Results

### 3.1. Selection of Pigment-Producing Endophytic Fungi from Fridericia chica

**Figure 1:**
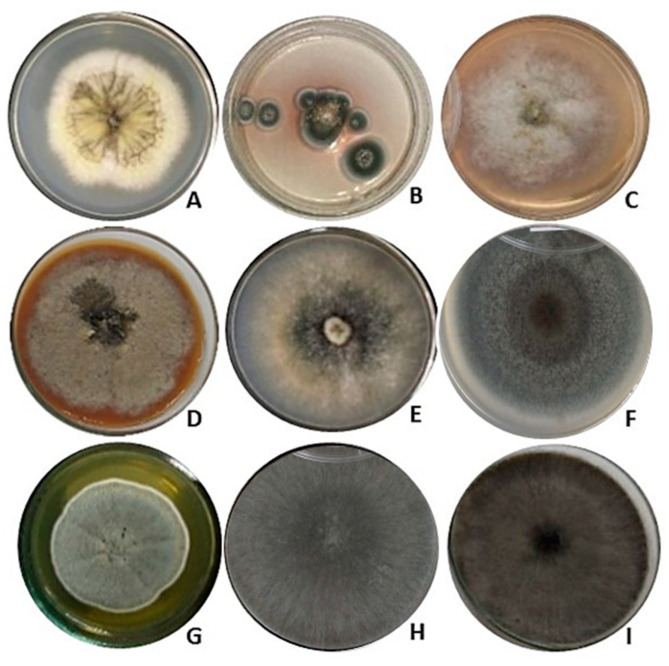
Endophytic fungi of *Fridericia chica* selected as pigment producers. (**A**) *Aspergillus welwitschiae* CG2-7, mycelium yellow pigment; (**B**) *A. sydowii* CF1-3, extracellular violet pigment; (**C**) *Diaporthe cerradensis* CG2-4, extracellular light orange pigment; (**D**) *Diaporthe cerradensis* CG2-12, extracellular caramel-colored pigment; (**E**) *Hypoxylon investiens* CF1-37, extracellular green pigment; (**F**) *Curvularia* sp. CF3-5, extracellular dark gray pigment; (**G**) *Penicillium rubens* CG2-5, extracellular yellow pigment; (**H**) *Neoscytalidium* sp. CG1-2, extracellular dark gray pigment, and (**I**) *Neoscytalidium* sp. CG2-10, extracellular dark gray pigment. Fungi cultivation was performed in 90x25 mm Petri dishes.

The 121 isolates of *F. chica* were grown in PDA. Of these, nine produced extracellular pigments of different colors diffused in the solid culture medium. The nine fungi were selected for cultivation in liquid media and for species identification via molecular analysis. The nine fungal colonies that produced pigments when grown in PDA medium are shown in Figure 1.

### 3.2. Pigment Production Using Submerged Fermentation

The extracts obtained from the submerged culture of the nine isolates were evaluated in terms of pigment production. After the growth of the fungi in two different culture media (14 days, static, at 30 °C), the metabolic broths were extracted and analyzed regarding the maximum absorbance wavelength of the pigment (λmax) between 400 and 700 nm. The pigmented fungal extracts that stood out were those obtained from the cultivation of the isolates *Hypoxylon investiens* CF1-37 (UA_700_ = 0.550), *Neoscytalidium* sp. CG2-10 (UA_400_ = 1.96) and *Penicillium rubens* CG2-5 (UA_400_ = 1.74) (Table 2 and Table 3). 

In Table 2 and Table 3, it is observed that fungi grown in the medium containing yeast extract (YEX) were able to produce more pigments than when grown in the medium containing peptone (PEP). Most extracts obtained the maximum absorbance at 700 nm.

### 3.3. Phylogenetic Analysis of Pigment-Producing Endophytic Fungi from Fridericia chica

For isolates CF1-3 and CG2-7 belonging to the *Aspergillus* genus, the phylogenetic tree was constructed based on the concatenated sequences of ITS + *tub*2 + *cal* + *rpb*2 and the best-fitting model according to the Bayesian information criterion (BIC) was TNe + I + G4: part1, TIM2 + F + G4: part2, TNe + G4: part3 and HKY + F + I + G4: part4. A total of 59 representative isolates of the genus *Aspergillus* (Supplementary Table S1) were analyzed and the results showed that CF1-3 belongs to the species *A. sydowii* (CBS 593.65) and CG2-7 to *A. welwitschiae* (CBS 139.54), with high bootstrap support (Supplementary Figure S1).

For the *Penicillium* isolate CG2-5, the phylogenetic tree was constructed based on the combined *tub*2 + *rpb*2 sequences and the best-fitting model according to the BIC was TNe + G4: part1 and TNe + G4: part2. We analyzed 37 representative isolates of the genus *Penicillium* (Supplementary Table S1) and the results of the comparison suggest that this isolate is phylogenetically related to the species *P. rubens* (DTO 98E8—Ex-lectotype), with a bootstrap of 99% (Supplementary Figure S2). 

The phylogenetic tree for the genus *Neoscytalidium* was constructed based on the concatenated analysis of ITS + *tub*2 + *tef*1, and the best-fitting model according to the BIC was TI-Me + G4: part1, HKY + F + G4: part2 and TN + F + G4: part3. A total of 52 representative isolates (Supplementary Table S1) were analyzed and the results of the phylogenetic comparison indicate that isolates CG1-2 and CG2-10 belong to this genus. Based on the observation of the phylogenetic tree, we can suggest an evolutionary proximity to *N. oculi*. However, due to the limitations of the available data, more information should be collected and analyzed to resolve the polytomy that was observed at this branching point between these lineages (Supplementary Figure S3).

The phylogenetic tree for the genus *Diaporthe* was constructed based on the combined sequences ITS + *cal* + *tef*1, and the best-fitting model according to the BIC was HKY + F + I + G4: part1, TIM2e + I + G4: part2 and HKY + F + I + G4: part3. A total of 75 representative isolates were included for this genus (Supplementary Table S1) and, from the analysis, we can suggest that the isolates CG2-4 and CG2-12 belong to the species *Diaporthe cerradensis* (Supplementary Figure S4). 

The phylogenetic structure elaborated for the CF3-5 isolate was constructed from the combined sequences ITS + *rpb*2, and the best-fitting model according to the BIC was TNe + I + G4: part1 and TIMe + I + G4: part2. In this analysis, we chose to combine only two loci, since, when examining the taxonomy on the GenBank platform, it was noted that, for the genus *Curvularia*, in addition to the loci used in this study, others are often used for molecular identification. LSU (large subunit gene of nuclear ribosomal RNA), EF1a (elongation factor 1-alpha) and GAPDH (glyceraldehyde-3-phosphate dehydrogenase) are used for phylogenetic analysis.

We included 68 representative isolates for the genus *Curvularia* (Supplementary Table S1) and, based on the observations, the isolate CF3-5 grouped with species *Curvularia chiangmaiensis* (CPC 28829), *C. dactylocteniicola* (CPC 28810), *C. lunata* (CBS 730.96), *C. sorghina* (BRIP 15900) and *C. tropicalis* (BRIP 14834), indicating that the utilized barcodes are insufficient for phylogenetic resolution (Supplementary Figure S5).

For the isolate CF1-37, the phylogenetic analysis was constructed from the combined sequences ITS + *tub*2, and the best-fitting model according to the BIC was TIM2e + I + G4: part1 and GTR + F + I + G4: part2. In all, 91 representative isolates of the genus *Hypoxylon* (Supplementary Table S1) were analyzed, and the results of the comparison suggest that this isolate is related to the *H. investiens* strain (MUCL 53307), with a 100% bootstrap (Supplementary Figure S6).

The sequences obtained in this study were deposited in GenBank (Table 4).

### 3.4. Biological Activities of the Most-Promising Pigmented Extract

The pigmented extract produced by the endophytic fungus *Hypoxylon investiens* CF1-37 was evaluated in terms of its antioxidant and antimicrobial activities. The results are presented in Table 5.

As shown in Table 5, at a concentration of 10 mg/mL, the pigmented extract of *H. investiens* CF1-37 demonstrates a remarkable antioxidant potential (AA) of 91.08%, which highlights the ability of the metabolites generated by this extract to sequester DPPH•. free radicals. The EC_50_ was calculated from a calibration curve y = 77.393 x − 10.066 (R^2^ = 0.988). Regarding the FRAP assays, a promising result of 105.54 µmol TE/g was observed for the antioxidant potential of the pigmented extract of *H. investiens* CF1-37. The FRAP results were obtained from a calibration curve (y = 0.0034 x − 0.0448, R^2^ = 0.9927) using Trolox (0–140 µM) and expressed in Trolox equivalents (TEs) per gram of extract.

The pigmented extract from *H. investiens* CF1-37 proved effective in inhibiting the growth of Gram-negative bacteria (*E. coli*, *S. marcescens*, *P. mirabilis* and *K. pneumoniae*) as well as fungal strains (*C. albicans* and *C. tropicalis*).

### 3.5. Chemical Profile of the Most-Promising Pigmented Extract

**Figure 2:**
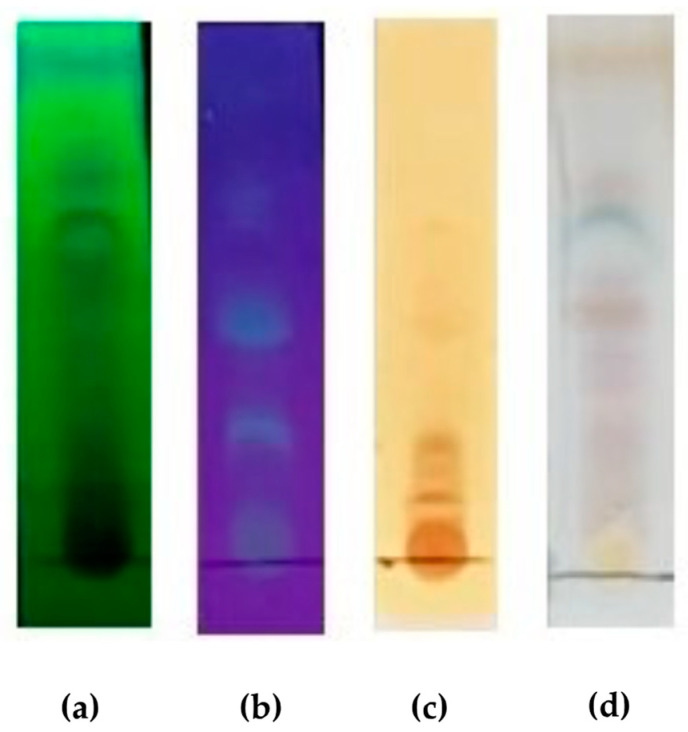
Thin layer chromatography of the pigmented extract produced by the endophytic fungus *Hypoxylon investiens* CF1-37, isolated from *Fridericia chica*. (**a**) UV light at 254 nm, indicating the presence of conjugated double bonds; (**b**) stained with aluminum chloride and exposure under UV light at 365 nm, indicating the presence of flavonoids; (**c**) stained with ferric chloride, indicating the presence of phenolic compounds (brown spots); and (**d**) stained with vanillin/H_2_SO_4_, indicating the presence of terpenoids (light pink spots). TLC aluminum sheets (Macherey-Nagel, 20 × 20 cm, silica gel 60 matrix, fluorescent indicator) was used.

The pigmented extract produced by *H. investiens* CF1-37 was analyzed using TLC to identify the chemical classes present in the active extract. The visualization of the chromatographic plate under UV light at 254 nm revealed the presence of conjugated double bonds (Figure 2a). The fluorescence observed when the plate was treated with aluminum chloride and exposed to UV light at 365 nm indicates the presence of flavonoids in the pigmented extract (Figure 2b). Additionally, the brown spot observed when the plate was developed with ferric chloride is indicative of the presence of phenolic compounds (Figure 2c). Further development of the plate with vanillin/H_2_SO_4_ showed a purple coloration, indicating the presence of terpenoids (Figure 2d). It is noteworthy that alkaloids were not detected in the pigmented active extract when using TLC. 

**Figure 3:**
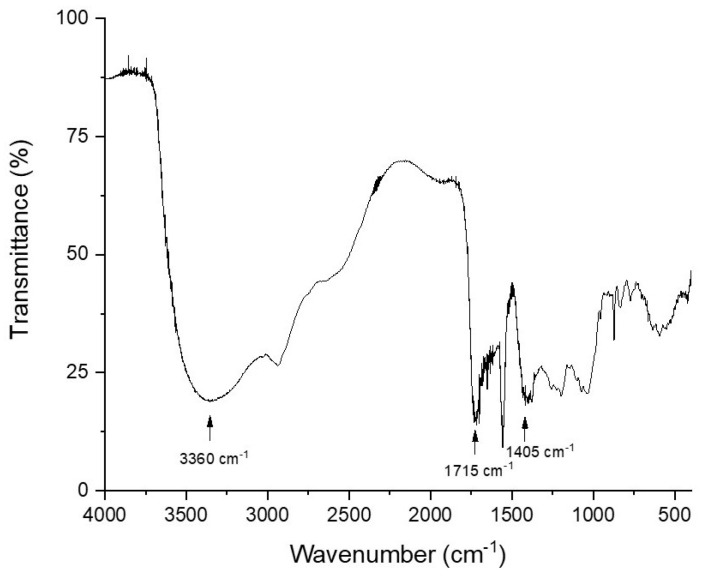
Fourier-transform infrared spectrum (FTIR) of the pigmented active extract produced by the endophytic fungus *Hypoxylon investiens* CF1-37, isolated from *Fridericia chica* leaves.

The infrared spectrum obtained from the pigmented extract produced by the endophytic fungus *H. investiens* CF1-37 is shown in Figure 3. The active extract presented an expressive band at 3360 cm^−1^*,* which seems to expand from 3700 to 2100 cm^−1^, with possible other bands appearing at 2550 cm^−1^ and at 2950 cm^−1^. Other relevant bands to be highlighted appear at 1715 cm^−1^ and 1405 cm^−1^, in addition to several other bands in the region of 400 to 1852 cm^−1^. These bands are expected for an extract that is rich in phenolic compounds and flavonoids, and this is a result that is also confirmed by the TLC.

## 4. Discussion

The isolation of endophytic fungi associated with medicinal plants is of great importance due to their ability to synthesize secondary metabolites similar to the host, which presents great potential for the discovery of new bioactive compounds [46]. *F. chica* is a medicinal plant that has been extensively investigated regarding its chemical composition. It has been observed that the red coloration of the extract, its striking characteristic, is related to the presence of 3-deoxyanthocyanidins, denominated carajurine (6,7-dihydroxy-5,4’-dimethoxyflavilium) and carajurone (6,7,4’-trihydroxy-5-methoxyflavilium) [17,18]. However, to date, only one study (by Gurgel et al. [8]) has investigated the potential of endophytic fungi associated with this medicinal plant.

Fungi are recognized as a viable and readily available alternative for obtaining natural pigments. In addition, they have advantages in relation to plants, as they can produce pigments regardless of the season, exhibit easy and fast growth in accessible culture media, generate pigments with different shades that are more stable and soluble, and are simpler to process [43]. These organisms can also provide health benefits such as anticancer activity, antimicrobial activity and antioxidant activity [10].

In this study, only the extracellular production of pigments was evaluated and the selection of media was performed based on the existing literature available for the production of pigments by filamentous fungi in liquid cultures. The AcOEt extracts obtained from the liquid culture of the selected isolates were analyzed with a UV/VIS spectrophotometer to obtain the wavelength of the maximum absorbance (λ) of the pigment. A variation in the coloration of the extracts obtained in culture containing peptone was observed when compared to those containing yeast extract, depending on the species, which was confirmed with the reading of the absorbances in the spectrophotometer (Table 2 and Table 3). This demonstrates that, depending on the species selected, some nitrogen sources can be more easily assimilated and promote higher yields of the desired product [47]. The addition of various nitrogen sources, such as ammonium, peptone and others, is associated with an increased pigment yield, modification of the tone of the fermentation liquid and improved light stability of pigments in species of the genus *Monascus* [48].

The nine isolates of *F. chica* selected were described based on molecular analysis and belong to the genera and species *Aspergillus welwitschiae* CG2-7, *A. sydowii* CF1-3, *P. rubens* CG2-5, *Neoscytalidium* sp. CG1-2 and CG2-10, *Diaporthe cerradensis* CG2-4 and CG2-12, *Curvularia* sp. CF3-5, and *Hypoxylon investiens* CF1-37. All the loci used in this study provided acceptable information on the species identification and the sequences obtained were submitted to NCBI GenBank with their respective accession numbers (Table 4).

The isolates *Aspergillus welwitschiae* CG2-7 and *A. sydowii* CF1-3 belong to the genus *Aspergillus*, family Aspergillaceae, order Eurotiales and class Eurotiomycetes. In this genus, some of the species are already described as potential producers of pigments. Several members of the genus *Aspergillus*, such as *A. niger*, are known to synthesize a wide variety of pigments, such as aspergillin, asperenone, azaphilones (azanigerones A–F), and melanin. Others, such as *A. nidulans, A. glaucus, A. versicolor,* and *A. purpureus*, are reported to produce yellow and red pigments [48].

With AcOEt, it was possible to extract a yellowish color from the culture of *A. welwitschiae* CG2-7 in a medium containing yeast extract, while from the culture of *A. sydowii* CF1-3, it was not possible to obtain a colored extract using this solvent. The extraction of the pigment produced by *A. sydowii* CF1-3 was not efficient and this was probably due to the choice of solvent (ethyl acetate). In their study with eight fungal strains, which included *A. sydowii*, Da Costa Souza et al. [49] also failed to extract pigments with this solvent and suggested that the pigments of this lineage could be insoluble in ethyl acetate. Only *A. sydowii* was reported as a pigment producer in the study by Da Costa Souza et al. [49]. No reports of this type of study with *A. welwitschiae* were found.

The strain CG2-5 of *P. rubens* produced an extracellular yellow color in the solid and liquid culture medium (only in the medium with yeast extract) and was selected as a potential pigment producer. It is important to note that in the literature there are no reports of pigment production by this lineage; thus, this is the first report of pigment production by this species, which should be further investigated. 

The genus *Penicillium*, family Aspergillaceae, order Eurotiales and class Eurotiomycetes, is widely recognized as a rich source of bioactive secondary metabolites. Studies demonstrate that this genus is a potent producer of pigments, such as the arpink redTM (first commercial red dye) produced by *P. oxalicum* [48]. Many studies have focused on investigating the optimization of the culture parameters for pigment production. Species of *Penicillium* are widely studied, such as *P. convolutum*, *P. flavigenum*, *P. mallochii*, *P. melinii*, *P. oxalicum*, *P. purpureogenum P. simplicissimum* and *P. sclerotiorum* [48,50,51,52].

Strains CG1-2 and CG2-10 were isolated from different *F. chica* trees, and both of these strains were found in the branches. The analysis of the combined loci suggests that these isolates can be characterized as belonging to the genus *Neoscytalidium*, family Botryosphaeriaceae, order Botryosphaeriales, and class Dothideomycetes. This new genus described in 2006 by Crous and Slippers [53] currently has seven valid species. When compared to these species, isolates CG1-2 and CG2-10 are closely related to *N. oculi*, with bootstrap support of 91%, whose sister group is *N. dimidiatum* (Supplementary Figure S3). 

The genus *Neoscytalidium* is often cited as a plant pathogen [54]. Both isolates CG1-2 and CG2-10 exhibited a dark mycelium, which led to their selection for submerged culture. However, the coloration was not visually prominent during this culture, both in the yeast extract medium and in the peptone medium. It is relevant to note that in the literature, there are no records of studies considering this fungal genus as a pigment producer. In addition, we must take into account the fact that due to its general classification as a pathogen, its viability for pigment production and industrial use may be limited.

The genus *Diaporthe* (syn. *Phomopsis*) belongs to the family Diaporthaceae, order Diaporthales, in the class Sordariomycetes, and is widely distributed geographically. It comprises pathogens, endophytes and saprobes that affect a wide range of hosts [55,56]. The isolates CG2-4 and CG2-12 were identified as belonging to the species *Diaporthe cerradensis*, showing 100% similarity with the accession sequences (UFM-GCB4807, CMRP4324, LGMF1616 and CMRP4331) for the combined analysis of the barcodes ITS, *cal* and *tef*1, which are loci that are commonly used for the identification of species of *Diaporthe* [55,56,57,58]. 

The species *D. cerradensis* was described in the study by Iantas et al. [58] as one of the species of this genus that shows potential against plant pathogens, mainly against the pathogenic fungi *Colletotrichum abscissum* and *Phyllosticta citricarpa*, which attack citrus fruits. In our study, we found that the isolates from *D. cerradensis* presented a light brown hue in the solid PDA medium, which led to their selection for cultivation in the liquid medium. However, during submerged fermentation, it was not possible to observe the production of pigments by these isolates. To the best of our knowledge, there are no reports in the literature describing this species, or even this genus, as being promising in terms of pigment production. On the other hand, the genus *Diaporthe* is a rich source of bioactive secondary metabolites with antimicrobial and antifungal activities [57,58,59,60].

The isolate CF3-5 was identified as belonging to the genus *Curvularia*, family Pleosporaceae, order Pleosporales and class Dothideomycetes. This genus includes phytopathogenic species with a global distribution and a wide variety of hosts, especially cereals and grasses (Poaceae) [58]. In the solid medium, this isolate presented a dark mycelium, and it was subsequently chosen for the submerged culture, in which it produced a yellowish coloration, both in the medium with yeast extract and in the medium with peptone. In the spectrophotometric analyses, absorbances were recorded in the wavelength range of 700 nm (Table 2 and Table 3). 

In a study conducted by Sharma et al. [60], the extraction of pigments from *Trichoderma virens, Alternaria alternata* and *C. lunata* was explored for textile dyeing. The three fungi showed great potential as sources of dyes for dyeing fabrics, and it was found that these pigments did not present toxicity to human skin. Thus, we can suggest that most of the species identified in our study may constitute promising sources of dyes and/or metabolites with biotechnological applications.

Other studies, such as that by Da Costa Souza et al. [49], examined extracellular pigment production in different fungal strains of distinct species and identified these strains based on sequences of the internal transcribed spacer. The authors identified *A. sydowii* CML2967, *A. aureolatus* CML2964, *A. keveii* CML2968, *P. flavigenum* CML2965, *P. chermesinum* CML2966, *Epicoccum nigrum* CML2971, *Lecanicillium aphanocladii* CML2970 and *Fusarium* sp. CML2969, which have shown promise as new pigment sources with important industrial applications. Bouhri et al. [51] studied the red–orange pigment-producing strain *P. mallochii* TACB-16, identified via molecular methods (analysis of the ITS region). The authors analyzed the maximum absorption wavelength (λmax) of the pigment of *P. mallochii,* which was determined to be 450 nm. In addition, the results showed that the fungal pigment was resistant to different temperatures and pH values and also demonstrated that the pigment had antiproliferative effects on the T98G cell line (glioblastoma cell lines).

The phylogenetic inference of isolate CF1-37 showed high support with *H. investiens* (MUCL 53307). Belonging to the Hypoxylaceae family, it stands out for its diversity in terms of the production of secondary metabolites, such as antiparasitic agents, enzyme inhibitors, immunomodulators, antimicrobial substances or pigments, mainly extracted from its fruiting stages [61,62]. In submerged culture, this isolate showed a green coloration, which was extracted with ethyl acetate in both liquid cultures performed in this study. The absorbance reading revealed values present in the red absorption range (Table 2 and Table 3). 

A number of genera belonging to the Xylariaceae family, including *Daldinia, Hypoxylon*, *Jackrogersella* and others, have a significant ability to produce pigments with a wide variety of colors and shades. Some species of the genus *Hypoxylon* have already been reported as pigment producers, for example, *H. fragiforme* (hypoxyxylerone, cytochalasin H, fragiformins A–B and mitorubrine), *H. howeanum* (mitorubrine and azaphilones), *H. lechatii* (vermelhotin and hypoxyvermelhotins A–C), *H. fuscum* (daldinin A–C), *H. fulvo-sulphureum* (derivatives of mitorubrinol), *H. sclerophaeum* (hypoxilone), *H. rickii* (rickenyl B and D), *H. lenormandii* and *H. jaklitschii* (lenormandines A–G) and *H. rubiginosum* (mitorubrine, rubiginosine and hypomyltin) [48,60]. The diversity and complexity of the secondary metabolites in this subfamily (Xylariaceae: Hypoxyloideae) have been demonstrated through several studies [60], but no reports have been found in the literature on the production of extracellular pigment by *H. investiens*. 

The pigmented extract of *H. investiens* CF1-37 showed antioxidant and antimicrobial activity. In a recent study conducted by our group [8], we analyzed an extract of *H. investiens* CF1-37 obtained under other cultivation conditions and the results were similar to those observed in this study. In the antioxidant analyses, the values of AA = 93.68% were observed for the DPPH• free radical sequestration, and FRAP = 171.1 µmol TE/g, while for the strains tested, the extract inhibited the growth of *S. aureus, C. albicans, C. tropicalis* and *C. parapsilosis*. No other studies analyzing the antioxidant and antimicrobial potential of this species were found. 

The results of the TLC of the pigmented extract produced by the endophytic fungus *H. investiens* CF1-37 indicated the presence of phenolic compounds and flavonoids. Phenolic compounds are a class of molecules that have a wide distribution in natural products and have attracted a lot of attention due to their antioxidant and antimicrobial properties [63]. Antioxidant compounds produced by endophytic fungi are usually from the class of phenolic compounds, such as flavonoids and phenolic acids, which corroborates the results found in this study [64,65,66]. Additionally, the infrared spectrum obtained for the active pigmented extract is in accordance with the results of the TLC. The band at 3360 cm^−1^ is probably associated with O–H stretching vibrations, while the band at 1701 cm^−1^ is probably related to a C=O stretching of the flavonoids. These bands were also observed by Oliveira et al. [67], who analyzed the extracts of several plants that were rich in phenolic compounds and flavonoids, and by Falcão et al. [63], who analyzed an extract rich in phenolic compounds produced by the endophytic fungus *Aspergillus niger* MgF2.

## 5. Conclusions

This study provides preliminary information on the production of extracellular pigments by endophytic fungi isolated from the Amazonian species *F. chica*. A total of 121 endophytic fungi were isolated from this medicinal plant, and nine showed potential for pigment production. Molecular identification and phylogenetic analysis of these nine strains revealed the species *Aspergillus welwitschiae*, *A. sydowii*, *Diaporthe cerradensis* (2 strains), *Hypoxylon investiens* and *Penicillium rubens*. The genera *Neoscytalidium* sp. (2 strains) and *Curvularia* sp. were also identified.

Among these strains, *H. investiens* CF1-37, *Neoscytalidium* sp. CG2-10, and *P. rubens* CG2-5 stood out as the best producers of colored compounds. The pigment production capabilities varied depending on the fungal species tested, and this could be influenced by the composition of the culture medium. The culture medium containing yeast extract seemed to favor the production of these metabolites.

*H. investiens* CF1-37 produced a green extract that exhibited antioxidant and antimicrobial activity. The chemical profile if this pigmented extract showed the presence of phenolic compounds and flavonoids. In future studies, the pigment production by this isolate should be optimized. In addition, the pigment molecule should be isolated and identified. In this study, we demonstrated the potential of *H. investiens* CF1-37 as a new source of pigments for industrial applications.

## Figures and Tables

**Table 1:** Description of the combined markers used for amplification and sequencing, aiming at the identification of pigment-producing endophytic fungi from *Fridericia chica*.

Locus ^1^	Primer	Sequence 5′-3′	T (°C) ^2^	Reference
ITS	*Its*1	TCCGTAGGTGAACCTGCGG	62	[37]
	*Its*4	TCCTCCGCTTATTGATATGC		
*tub*2	Bt-2a	GGT AAC CAA ATC GGT GCT GCT TTC	62	[38]
	Bt-2b	ACC CTC AGT GTA GTG ACC CTT GGC		
*cal*	CALM-228F	GAGTTCAAGGAGGCCTTCTCCC	54	[39]
	CALM-737R	CATCTTTCTGGCCATCATGG		
*tef*1	EF1-728F	CAT CGA GAA GTT CGA GAA GG	58	[39]
	EF1-986R	TAC TTG AAG GAA CCC TTA CC		
*rpb*2	RPB2-6F	TGG GGK WTG GTY TGY CCT GC	58	[40]
	fRPB2-7cR	CCC ATR GCT TGY TTR CCC AT		

^1^ ITS: internal transcribed spacer; *tub*2: partial beta-tubulin gene; *cal*: calmodulin; *tef*1: partial elongation factor 1-alpha gene; *rpb*2: second largest protein subunit of DNA-directed RNA polymerase II. ^2^ T (°C): annealing temperature.

**Table 2:** Pigment production by endophytic fungi from *Fridericia chica* in submerged fermentation, cultured in a medium containing peptone (PEP) as the nitrogen source. The maximum absorbance values (λmax) of the pigmented fungal extracts were obtained from a scan between 400 and 700 nm using a UV/VIS spectrophotometer.

λ (nm)	Endophytic Fungi from *Fridericia chica*								
	CG2-7	CF1-3	CG2-4	CG2-12	CF1-37	CG2-10	CG2-5	CG1-2	CF3-5
400	0	0	0	0	0	0	0	0	0
450	0	0	0	0	0	0	0	0	0
550	0	0.070	0	0	0	0	0.200	0	0
580	0	0.070	0	0	0	0	0.210	0	0
600	0	0.090	0	0	0	0	0.220	0	0
700	0	0.340	0.220	0.030	0	0.04	0.450	0	0.170
Pigmented extract	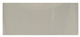	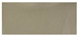	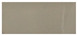	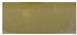	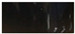	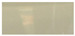	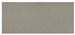	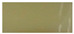	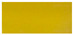

**Table 3:** Pigment production by endophytic fungi from *Fridericia chica* in submerged fermentation, cultured in a medium containing yeast extract (YEX) as the nitrogen source. The maximum absorbance values (λmax) of the pigmented fungal extracts were obtained from a scan between 400 and 700 nm using a UV/VIS spectrophotometer.

λ (nm)	Endophytic Fungi from *Fridericia chica*								
	CG2-7	CF1-3	CG2-4	CG2-12	CF1-37	CG2-10	CG2-5	CG1-2	CF3-5
400	0	0	0	0	0	1.96	1.74	0	0
450	0	0	0	0	0	0.610	0.330	0	0.110
550	0	0.020	0	0	0	0.930	0	0.530	0.150
580	0	0.030	0	0	0	0.950	0	0.530	0.130
600	0	0.040	0.020	0	0.480	0.940	0	0	0.140
700	0.120	0.300	0.260	0.120	0.550	1.22	0.040	0	0.380
Pigmented extract	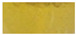	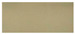	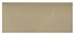	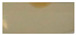	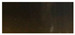	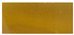	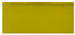	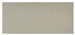	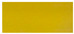

**Table 4:** GenBank accession numbers for the fungal isolates from *Fridericia chica* that produce pigments. Newly deposited sequences are shown in bold.

Isolate	Species	Source	GenBank Accession Number				
			ITS	*tub*2	*cal*	*tef*1	*rpb*2
CF1-3	*Aspergillus sydowii*	*F. chica*	**OR799521**	**OR805272**	**OR805277**	-	**OR805285**
CG2-7	*Aspergillus welwitschiae*	*F. chica*	**OR799615**	**OR805273**	**OR805278**	-	**OR805286**
CF3-5	*Curvularia* sp.	*F. chica*	**OR799998**	-	-	-	**OR805287**
CG2-4	*Diaporthe cerradensis*	*F. chica*	**OR800272**	-	**OR805279**	**OR805281**	-
CG2-12	*Diaporthe cerradensis*	*F. chica*	**OR800278**	-	**OR805280**	**OR805282**	-
CF1-37	*Hypoxylon investiens*	*F. chica*	**OR800289**	**OR805274**	-	-	-
CG2-5	*Penicillium rubens*	*F. chica*	-	**OR805275**	-	-	**OR805288**
CG1-2	*Neoscytalidium* sp.	*F. chica*	**OR800290**	-	-	**OR805283**	-
CG2-10	*Neoscytalidium* sp.	*F. chica*	**OR800291**	**OR805276**	-	**OR805284**	-

- = no resolution. ITS = internal transcribed spacer region. *tub*2 = β-tubulin. *cal* = calmodulin. *tef*1 = partial elongation factor 1-alpha gene. *rpb*2 = second largest protein subunit of DNA-directed RNA polymerase II.

**Table 5:** Antioxidant activity and antimicrobial activity of the pigmented extract produced by the endophytic fungus *Hypoxylon investiens* CF1-37, isolated from *Fridericia chica* leaves. Antioxidant activity (AA) obtained using the DPPH method, the effective concentration for the sequestration of 50% of the DPPH• free radicals (EC_50_), and the ferric reducing antioxidant power (FRAP) method. Antimicrobial activity determined via the microdilution technique.

Antioxidant Activity *	AA (%)		EC_50_ (μg/mL)				FRAP (µmol TE/g)			
*H. investiens* CF1-37 extract	91.08		780				105.54			
Quercetin	98.00		8				NT			
Ascorbic acid	NT		NT				163.10			
**Antimicrobial Activity**	**MIC (mg/mL)**									
	EC	SM	PM	PA	BS	SA	KP	SE	CA	CT
*H. investiens* CF1-37 extract	5.00	5.00	2.50	1.25	-	-	2.50	-	2.50	2.50

* = assays carried out with the fungal extract at a concentration of 10 mg/mL. EC = *Escherichia coli*; SM = *Serratia marcescens*; PM = *Proteus mirabilis*; PA = *Pseudomonas aeruginosa*; BS = *Bacillus subtilis*; SA = *Staphylococcus aureus*; KP = *Klebsiella pneumoniae*; SE = *Salmonella enterica*; CA = *Candida albicans*; CT = *Candida tropicalis*; “-” = no antimicrobial activity; NT = not tested. Levofloxacin was used as the positive control for bacterial strains. MICs (μg/mL): EC = 0.05; SM = 0.25; PM = 0.25; PA = 0.50; BS = 0.50; SA = 0,50; KP = 4.00; SE = 0.25. Terbinafine was used as the positive control for fungal strains. MICs (μg/mL): CA = 6.25; CT = 6.25.

## Data Availability

Data are contained within the article and supplementary materials.

## Supplementary Materials

The following supporting information can be downloaded at https://www.mdpi.com/article/10.3390/jof10010077/s1. Table S1. GenBank accession numbers of sequences used in the phylogenetic analyses of multiple genes. Figure S1. Concatenated phylogenetic analysis by Maximum Likelihood (ML) of the endophytic fungi CF1-3, isolated from *Fridericia chica* leaves, and CG2-7, isolated from *F. chica* branches, based on partial sequences of ITS, *tub*2, *cal* and *rpb*2. The scale bar of 0.07 represents the number of changes and the number indicates the bootstrap support of the branches. The sequences of the isolates studied here are highlighted in bold. The tree was rooted in *Hamigera avellanea* NRRL 1938. Figure S2. Concatenated phylogenetic analysis by Maximum Likelihood (ML) of the endophytic fungus CG2-5, isolated from *Fridericia chica* branches, based on partial sequences of *tub*2 and *rpb*2. The scale bar of 0.08 represents the number of changes and the number indicates the bootstrap support of the branches. The sequences of the isolates studied here are highlighted in bold. The tree was rooted in *Aspergillus glaucus* NRRL 116. Figure S3. Concatenated phylogenetic analysis by Maximum Likelihood (ML) of the endophytic fungi CG1-2 and CG2-10, both isolated from branches of *Fridericia chica*, based on partial sequences of ITS, *tub*2 and *tef*1. The scale bar of 0.03 represents the number of changes and the number indicates the bootstrap support of the branches. The sequences of the isolates studied here are highlighted in bold. The tree was rooted in *Aplosporella hesperidica* CBS 732.79. Figure S4. Concatenated phylogenetic analysis by Maximum Likelihood (ML) of the endophytic fungi CG2-4 and CG2-12, both isolated from branches of *Fridericia chica*, based on partial sequences of ITS, *cal* and *tef*1. The scale bar of 0.07 represents the number of changes and the number indicates the bootstrap support of the branches. The sequences of the isolates studied here are highlighted in bold. The tree was rooted in *Diaporthella corylina* CBS 121124. Figure S5. Concatenated phylogenetic analysis by Maximum Likelihood (ML) of the endophytic fungus CF3-5, isolated from the leaves of *Fridericia chica*, based on partial sequences of ITS and *rpb*2. The scale bar of 0.03 represents the number of changes and the number indicates the bootstrap support of the branches. The sequences of the isolates studied here are highlighted in bold. The tree was rooted in *Alternaria alternata* CBS 916.96. Figure S6. Concatenated phylogenetic analysis by Maximum Likelihood (ML) of the endophytic fungus CF1-37, isolated from the leaves of *Fridericia chica*, based on partial sequences of ITS and *tub*2. The scale bar of 0.5 represents the number of changes and the number indicates the bootstrap support of the branches. The sequences of the isolates studied here are highlighted in bold. The tree was rooted in *Biscogniauxia nummularia* MUCL 51395.

## References

[1] Dhyani A., Jain R., Pandey A. (2019). Contribution of root-associated microbial communities on soil quality of oak and pine forests in the Himalayan ecosystem. Trop. Eco..

[2] Bacon C.W., White J.F. (2000). Microbial Endophytes.

[3] Rathod D., Dar M.G.A., Shrivastava R.B., Rai M., Varma A., Chandra S., Lata H., Varma A. (2013). Microbial Endophytes: Progress and Challenges. Biotechnology for Medicinal Plants.

[4] Hamilton C.E., Bauerle T.L. (2012). A new currency for mutualism?. Fungal endophytes alter antioxidant activity in hosts responding to drought. Fungal Div..

[5] Fadiji A.E., Babalola O.O. (2020). Elucidating mechanisms of endophytes used in plant protection and other bioactivities with multifunctional prospects. Front. Bioeng. Biotechnol..

[6] Balkrishna A., Joshi R., Arya A.D.V., Wu Q.-S., Zou Y.-N., Xu Y.-J. (2022). Endophytic fungi: A Comprehensive Review on Their Secondary Metabolites, Pharmacological Interventions and Host Plant Interactions. Endophytic Fungi: Biodiversity, Antimicrobial Activity and Ecological Implications.

[7] Strobel G. (2018). The emergence of endophytic microbes and their biological promise. J. Fungi.

[8] Gurgel R.S., Pereira D.Í.M., Garcia A.V.F., Souza A.T.F., Silva T.M., Andrade C.P., Silva W.L., Nunez C.V., Fantin C., Procópio R.E.L. (2023). Antimicrobial and antioxidant activities of endophytic fungi associated with *Arrabidaea chica* (*Bignoniaceae*). J. Fungi.

[9] Pamphile J.A., Costa A.T., Rosseto P., Polonio J.C., Pereira J.O., Azevedo J.L. (2017). Biotechnological applications of secondary metabolites extracted from endophytic fungi: The case of *Colletotrichum* sp.. Rev. UNINGÁ.

[10] Sen T., Barrow C.J., Deshmukh S.K. (2019). Microbial pigments in the food industry—Challenges and the way forward. Front. Nutr..

[11] Anugraha A.C., Thomas T. (2021). A review on pigment producing soil fungi and its applications. Asian J. Mycol..

[12] Mapari S.A.S., Nielsen K.F., Larsen T.O., Frisvad J.C., Meyer A.S., Thrane U. (2005). Exploring fungal biodiversity for the production of water-soluble pigments as potential natural food colorants. Curr. Opin. Biotechnol..

[13] Mukherjee G., Mishra T., Deshmukh S.K., Satyanarayana T., Deshmukh S., Johri B. (2017). Fungal Pigments: An Overview. Developments in Fungal Biology and Applied Mycology.

[14] Dufosse L., Fouillaud M., Caro Y., Mapari S.A., Sutthiwong N. (2014). Filamentous fungi are large-scale producers of pigments and colorants for the food industry. Curr. Opin. Biotechnol..

[15] Gentry A.H. (2009). Bignoniaceae. Flora de Colombia no. 25.

[16] Cronquist A. (1981). An Integrated System of Classification of Flowering Plants.

[17] Batalha A.D.d.S.J., Souza D.C.d.M., Ubiera R.D., Chaves F.C.M., Monteiro W.M., da Silva F.M.A., Koolen H.H.F., Boechat A.L., Sartim M.A. (2022). Therapeutic potential of leaves from *Fridericia chica* (Bonpl.) L. G. Lohmann: Botanical aspects, phytochemical and biological, anti-inflammatory, antioxidant and healing action. Biomolecules.

[18] Schiozer A.L., Cabral E.C., Godoy L.A.F., Chaves F.C.M., Poppi R.J., Riveros J.M., Eberlinb M.N., Barata L.E.S. (2012). Electrospray ionization mass spectrometry fingerprinting of extracts of the leaves of *Arrabidaea chica*. J. Braz. Chem. Soc..

[19] Amaral R.R., Santos A.A.D., Saravia A., Botas G. (2012). Biological activities of *Arrabidaea chica* (Bonpl.) B. Verl. leaves. Lat. Am. J. Pharm..

[20] Siraichi J.T.G., Felipe D.F., Brambilla L.Z.R., Gatto M.J., Terra V.A., Cecchini A.L., Cortez L.E.R., Rodrigues-Filho E., Cortez D.A.G. (2013). Antioxidant capacity of the leaf extract obtained from *Arrabidaea chica* cultivated in Southern Brazil. PLoS ONE.

[21] Taffarelo D., Jorge M.P., Sousa I.M.d.O., Duarte M.C.T., Figueira G.M., Queiroz N.d.C.A., Rodrigues R.A.F., Carvalho J.E.d., Goes A.L.T.R., Foglio M.A. (2013). Activity of *Arrabidaea chica* (Humb. & Bonpl.) Verlot extracts obtained by biotechnological processes on fibroblast and human tumor cells. Quim. Nova.

[22] Ribeiro F.M., Volpato H., Lazarin-Bidóia D., Desoti V.C., Souza R.O.d., Fonseca M.J.V., Ueda-Nakamura T., Nakamura C.V., Silva S.d.O. (2018). The extended production of UV-induced reactive oxygen species in L929 fibroblasts is attenuated by posttreatment with *Arrabidaea chica* through scavenging mechanisms. J. Photochem. Photobiol. B Biol..

[23] Araújo W.L., Lima A.O.S., Azevedo J.L., Marcon J., Kuklinsky S.J., Lacava P.T. (2002). Manual: Isolamento de Microrganismos Endofíticos.

[24] Castellani A. (1939). Viability of some pathogenic fungi in distilled water. J. Trop. Med. Hyg..

[25] Heo Y.M., Kim K., Kwon S.L., Na J., Lee H., Jang S., Kim J.J. (2018). Investigation of filamentous fungi producing safe, functional water-soluble pigments. Mycobiol..

[26] Bose P., Gowrie S.U., Chathurdevi G. (2019). Optimization of culture conditions of growth and production of bioactive metabolites by endophytic fungus—*Aspergillus tamarii*. Int. J. Pharm. Biol. Sci..

[27] Zanette G.F. (2013). Pigmentos Naturais Produzidos por Fungos Filamentosos Isolados de *Theobroma grandiflorum* (Willd. Ex Spreng.) Schum. (cupuaçu). Ph.D. Thesis.

[28] Oliveira L.A. (2017). Produção, Isolamento e Identificação de Colorantes Produzidos por Fungos Isolados de Amostras do Solo amazônico. Master’s Thesis.

[29] Molyneux P. (2004). The use of the stable free radical diphenylpicrylhydrazyl (DPPH) for estimating antioxidant activity. Songklanakarin. J. Sci. Technol..

[30] Duarte-Almeida J.M., Dos Santos R.J., Genovese M.I., Lajolo F.M. (2006). Evaluation of the antioxidant activity using the β-carotene/linoleic acid system and the DPPH scavenging method. Food Sci. Technol..

[31] Benzie I.F.F., Strain J.J. (1996). The Ferric Reducing Ability of Plasma (FRAP) as a measure of “antioxidant power”: The FRAP assay. Anal. Biochem..

[32] (2017). Reference Method for Broth Dilution Antifungal Susceptibility Testing of Yeasts.

[33] Lopes J.L.C., Collins C.H., Braga G.L., Bonato P.S. (2006). Cromatografia em Camada Delgada. Fundamentos de Cromatografia.

[34] Wagner H., Bladt S. (1996). Plant Drug Analysis: A Thin Layer Chromatography Atlas.

[35] Doyle J., Doyle J.L. (1990). Isolation of plant DNA from fresh tissue. Focus.

[36] Oetari A., Rahmadewi M., Rachmania M.K., Sjamsuridzal W. (2018). Molecular identification of fungal species from deteriorated old chinese manuscripts in Central Library Universitas Indonesia. AIP Conf. Proc..

[37] White T.J., Bruns T., Lee S.J.W.T., Taylor J., Innis M.A., Gelfand D.H., Sninsky J.J., White T.J. (1990). Amplification and direct sequencing of fungal ribosomal RNA genes for phylogenetics. PCR Protocols: A Guide to Methods and Applications.

[38] Glass N.L., Donaldson G.C. (1995). Development of primer sets designed for use with the PCR to amplify conserved genes fromfilamentous ascomycetes. Appl. Environ. Microbiol..

[39] Carbone I., Kohn L.M. (1999). A method for designing primer sets for speciation studies in filamentous ascomycetes. Mycologia.

[40] Liu Y.J., Whelen S., Hall B.D. (1999). Phylogenetic relationships among ascomycetes: Evidence from an RNA polymerase II subunit. Mol. Biol. Evol..

[41] Hall T.A. (1999). BioEdit: A user-friendly biological sequence alignment editor and analysis, program for Windows 95/98/NT. Nucleic Acids Symp. Ser..

[42] Katoh K., Rozewicki J., Yamada K.D. (2019). MAFFT online service: Multiple sequence alignment, interactive sequence choice and visualization. Brief. Bioinform..

[43] Kumar S., Stecher G., Li M., Knyaz C., Tamura K. (2018). Mega X: Molecular Evolutionary Genetics Analysis across computingplatforms. Mol. Biol. Evol..

[44] Trifinopoulos J., Nguyen L.T., von Haeseler A., Minh B.Q. (2016). W-IQ-TREE: A fast online phylogenetic tool for maximum likelihood analysis. Nucleic Acids Res..

[45] Kalyaanamoorthy S., Minh B.Q., Wong T.K.F., Haeseler A.V., Jermiin L.S. (2017). ModelFinder: Fast model selection for accurate phylogenetic estimates. Nat. Methods.

[46] Kusari S., Lamshöft M., Zühlke S., Spiteller M. (2008). An endophytic fungus from *Hypericum perforatum* that produces hypericin. J. Nat. Prod..

[47] Lagashetti A.C., Dufossé L., Singh S.K., Singh P.N. (2019). Fungal pigments and their prospects in different industries. Microorganisms.

[48] Celestino J.D.R., Carvalho L.E., Lima M.P., Lima A.M., Ogusku M.M., Souza J.V.B. (2014). Bioprospecting of Amazon soil fungi with the potential for pigment production. Process Biochem..

[49] Da Costa Souza P.N., Grigoletto T.L.B., de Moraes L.A.B., Abreu L.M., Guimarães L.H.S., Santos C., Galvão L.R., Cardoso P.G. (2016). Production and chemical characterization of pigments in filamentous fungi. Microbiology.

[50] Méndez A., Pérez C., Montañéz J.C., Martínez G., Aguilar C.N. (2011). Red pigment production by *Penicillium purpurogenum* GH2 is influenced by pH and temperature. J. Zhejiang Univ. Sci. B.

[51] Bouhri Y., Askun T., Tunca B., Deniz G., Aksoy S.A., Mutlu M. (2020). The orange-red pigment from *Penicillium mallochii*: Pigment production, optimization, and pigment efficacy against Glioblastoma cell lines. Biocatal. Agric. Biotechnol..

[52] Oliveira L.A., Macedo M.M., Rodrigues J.L.S., Lima E.S., Hamill P.G., Dallas T.D., Souza J.V.B. (2021). Plant metabolite 5-pentadecyl resorcinol is produced by the Amazonian fungus *Penicillium sclerotiorum* LM 5679. Braz. J. Biol..

[53] Crous P.W., Slippers B., Wingfield M.J., Rheeder J., Marasas W.F.O., Phillips A.J.L., Alves A., Burgess T., Barber P., Groenewald J.Z. (2006). Phylogenetic lineages in the Botryosphaeriaceae. Stud. Mycol..

[54] Huang S.-K., Tangthirasunun N., Phillips A.J.L., Dai D.-Q., Wanasinghe D.N., Wen T.-C., Bahkali A.H., Hyde K.D., Kang J.-C. (2016). Morphology and phylogeny of *Neoscytalidium orchidacearum* sp. nov. (*Botryosphaeriaceae*). Mycobiology.

[55] Dissanayake A.J., Phillips A.J.L., Hyde K.D., Yan J.Y., Li X.H. (2017). The current status of species in *Diaporthe*. Mycosphere.

[56] Norphanphoun C., Gentekaki E., Hongsanan S., Jayawardena R., Manawasinghe I.S., Abeywickrama P.D., Bhunjun C.S., Hyde K.D. (2022). *Diaporthe*: Formalizing the species-group concept. Mycosphere.

[57] Gomes R.R., Glienke C., Videira S.I.R., Lombard L., Groenewald J.Z., Crous P.W. (2013). *Diaporthe*: A genus of endophytic, saprobic and plant pathogenic fungi. Persoonia.

[58] Iantas J., Savi D.C., Schibelbein R.d.S., Noriler S.A., Assad B.M., Dilarri G., Ferreira H., Rohr J., Thorson J.S., Shaaban K.A. (2021). Endophytes of Brazilian medicinal plants with activity against phytopathogens. Front. Microbiol..

[59] Marin-Felix Y., Senwanna C., Cheewangkoon R., Crous P.W. (2017). New species and records of *Bipolaris* and *Curvularia* from Thailand. Mycosphere.

[60] Sharma D., Gupta C., Aggarwal S., Nagpal N. (2012). Pigment extraction from fungus for textile dyeing. Indian J. Fibre Text. Res..

[61] Caro Y., Venkatachalam M., Lebeau J., Fouillaud M., Dufossé L., Merillon J.M., Ramawat K. (2016). Pigments and colorants from filamentous fungi. Fungal metabolites. Reference Series in Phytochemistry.

[62] Kuhnert E., Navarro-Muñoz J.C., Becker K., Stadler M., Collemare J., Cox R.J. (2021). Secondary metabolite biosynthetic diversity in the fungal family Hypoxylaceae and *Xylaria hypoxylon*. Stud. Mycol..

[63] Falcão L.D.S., Oliveira I.D.L., Gurgel R.S., de Souza A.T.F., Mendonça L.D.S., Usuda É.O., do Amaral T.S., Veggi P.C., Campelo P.H., de Vasconcellos M.C. (2024). Development of cassava starch-based films incorporated with phenolic compounds produced by an Amazonian fungus. Int. J. Biol. Macromol..

[64] Aguirre J.J., De La Garza T.H., Zugasti C.A., Belmares C.R., Aguilar C.N. (2013). The optimization of phenolic compounds extraction from cactus pear (*Opuntia ficus-indica*) skin in a reflux system using response surface methodology. Asian Pac. J. Trop. Biomed..

[65] Gubiani J.R., Habeck T.R., Chapla V.M., Silva G.H., Bolzani V.S., Araujo A.R. (2016). One strain-many compounds (OSMAC) method for production of phenolic compounds using *Camarops* sp., an endophytic fungus from *Alibertia macrophylla* (*Rubiaceae*). Quim. Nova.

[66] Dantas S.B.S., Moraes G.K.A., Araujo A.R., Chapla V.M. (2022). Phenolic compounds and bioactive extract produced by endophytic fungus. Coriolopsis rigida. Nat. Prod. Res..

[67] Oliveira R.N., Mancini M.C., de Oliveira F.C.S., Passos T.M., Quilty B., Thiré R.M.D.S.M., McGuinness G.B. (2016). FTIR analysis and quantification of phenols and flavonoids of five commercially available plants extracts used in wound healing. Rev. Mater..

