# Supplementary material for: Isolation and Identification of Pigment-Producing Endophytic Fungi from the Amazonian Species Fridericia chica
Source: J Fungi (Basel). 2024 Jan 19;10(1):77. doi: 10.3390/jof10010077 (PMC10821134; doi:10.3390/jof10010077)
Supplement: Supplementary file 1 [file jof-10-00077-s001.zip › jof-2782450-SI.pdf]

Supplementary Material

Table S1. GenBank accession numbers of sequences used in the phylogenetic analyses of multiple genes.

Species Name	Specimen No.	GenBank accession no.[1]				
		ITS	*tub2*	*cal*	*tef1*	*rpb2*
Aspergillus amoenus	NRRL 4838	EF652480	EF652304	EF652392	-	EF652216
Aspergillus angustatus	CBS 273.65	EU448283	AY339993	EU443984	-	KU867013
Aspergillus askiburgiensis	CCF 4716	LN873940	LN873952	LN873965	-	LN873984
Aspergillus asperescens	NRRL 4770	EF652475	EF652299	EF652387	-	EF652211
Aspergillus astellatus	NRRL 2396	EF652446	EF652270	EF652358	-	EF652182
Aspergillus aurantiobrunneus	CBS 465.65	KF465770	KF499567	-	-	KY006764
Aspergillus aurantiobrunneus	NRRL 4545	EF652465	EF652289	EF652377	-	-
Aspergillus aureolatus	NRRL 5126	EF652501	EF652325	EF652413	-	EF652237
Aspergillus austroafricanus	NRRL 233	JQ301891	JN853963	JN854025	-	JN853814
Aspergillus brasiliensis	CBS 101740	FJ629321	FJ629272	FN594543	-	KY006765
Aspergillus caespitosus	NRRL 1929	EF652428	EF652252	EF652340	-	EF652164
Aspergillus carbonarius	CBS 111.26	NR_111094	GU296700	FN594544	-	-
Aspergillus carbonarius	NRRL 369	EF661204	EF661099	EF661167	-	EF661068
Aspergillus costaricaensis	CBS 115574	NR_103604	GU296699	FN594545	-	-
Aspergillus creber	NRRL 58592	JQ301889	JN853980	JN854043	-	JN853832
Aspergillus cvjetkovicii	NRRL 227	EF652440	EF652264	EF652352	-	EF652176
Aspergillus ellipticus	CBS 70779	AY585548	AY585530	-	-	-
Aspergillus eucalypticola	53A2	EU482439	EU482435	EU482433	-	-
Aspergillus filifer	CBS 113636	EU448277	-	EU443973	-	KU866932
Aspergillus fructus	NRRL 239	EF652449	EF652273	EF652361	-	EF652185
Aspergillus griseoaurantiacus	DTO:267-D8	KJ775553	KJ775086	KJ775357	-	-
Aspergillus hongkongensis	HKU49	NR_138262	LC000552	LC000565	-	LC000578
Aspergillus ibericus	NRRL 35644	EF661200	EF661102	EF661163	-	-
Aspergillus jensenii	NRRL 58600	JQ301892	JN854007	JN854046	-	JN853835
Aspergillus luchuensis	KACC 46772	JX500081	JX500062	JX500071	-	-

Aspergillus neoniger	CBS 115656	FJ491682	FJ491691	FJ491700	-	-
Aspergillus neoniger	NRRL 62634	KC796401	KC796361	KC796377	-	-
Aspergillus niger	CBS 554.65	FJ629337	FJ629288	FN594540	-	-
Aspergillus niger	NRRL 326	EF661186	EF661089	EF661154	-	EF661058
Aspergillus oleicola	CBS 119.37	NR_135419	AY339996	EU443986	-	KU866923
Aspergillus pepii	AV11051B_IX	KU613368	KU613371	KU613365	-	-
Aspergillus piperis	CBS 112811	EU821316	FJ629303	FN594554	-	-
Aspergillus protuberus	NRRL 3505	EF652460	EF652284	EF652372	-	EF652196
Aspergillus pulverulentus	CBS 558.65	EU821317	HE984408		-	-
Aspergillus puulaauensis	NRRL 35641	JQ301893	JN853979	JN854034	-	JN853823
Aspergillus sclerotiicarbonarius	CBS 121057	EU159216	EU159229	EU159235	-	MN969091
Aspergillus sclerotioniger	CBS 115572	FJ629353	FJ629304	FN594557	-	-
Aspergillus spelunceus	NRRL 4989	EF652490	EF652314	EF652402	-	EF652226
Aspergillus stellatus	NRRL 1858	EF652426	EF652250	EF652338	-	EF652162
Aspergillus stellatus	NRRL 4793	-	EF652303	EF652391	-	-
Aspergillus subversicolor	NRRL 58999	JQ301894	JN853970	JN854010	-	-
Aspergillus sydowii	CBS 593.65	NR_131259	-	EU443971	-	-
Aspergillus tabacinus	NRRL 4791	EF652478	EF652302	EF652390	-	EF652214
Aspergillus tubingensis	NRRL 4875	EF661193	EF661086	EF661151	-	EF661055
Aspergillus undulata	CBS 261.88	EU448275	-	EU443989	-	KU866928
Aspergillus vadensis	CBS 113365	AY585549	AY585531	FN594560	-	-
Aspergillus venezuelensis	CBS 868.97	MH862679	AY339998	EU443977	-	KU866931
Aspergillus versicolor	ATCC 9577	KU729039	KU897001		-	-
Aspergillus versicolor	NRRL 13147	JQ301896	JN854003	JN854014	-	JN853803
Aspergillus versicolor	NRRL 13150	JQ301895	JN853976	JN854017	-	-
Aspergillus versicolor	NRRL 238	EF652442	EF652266	EF652354	-	EF652178
Aspergillus welwitschiae	CBS 139.54	FJ629340	FJ629291	KC480196	-	MN969100
Botryosphaeria agaves	MFLUCC 11-0125	JX646791	JX646841	-	JX646856	-
Botryosphaeria auasmontanum	CMW25413	KF766167	-	-	-	-
Botryosphaeria auasmontanum	MFLUCC 171071	MF398863	-	-	MF398915	-
Botryosphaeria corticis	CBS 119047	NR_111213	EU673107	-	-	-

Botryosphaeria corticis	CBS 119048	DQ299246	MT592464	-	-	-
Botryosphaeria dolichospermatii	NP1	MH491970	MH562327	-	MH491974	-
Botryosphaeria dothidea	CBS 115476	KF766151	-	-	-	-
Botryosphaeria dothidea	CMW8000	AY236949	AY236927	-	AY236898	-
Botryosphaeria fujianensis	NP57	MH491973	MH562330	-	MH491977	-
Botryosphaeria fusispora	MFLUCC 10-0098	JX646789	JX646839	-	JX646854	-
Botryosphaeria pseudoramosa	CERC 2001	KX277989	KX278198	-	KX278094	-
Botryosphaeria puerensis	CSF 6052	MT028569	MT028901	-	MT028735	-
Botryosphaeria qinlingensis	CFCC 52984	MK434301	-	-		-
Botryosphaeria ramosa	CMW26167	EU144055	-	-	EU144070	-
Botryosphaeria scharifii	IRAN 1529C	JQ772020	-	-	JQ772057	-
Botryosphaeria tenuispora	MUCC JPN 237	LC585278	LC585174	-	LC585150	-
Botryosphaeria wangensis	CERC 2298	KX278002	KX278211	-	KX278107	-
Botryosphaeria wangensis	CGMCC 3.18744	NR_159555		-		-
Cophinforma atrovirens	CBS 124934	MH863428	-	-	-	-
Cophinforma atrovirens	CMW22674	FJ888473	-	-	FJ888456	-
Cophinforma atrovirens	CMW54174	MT934421	MT862147	-	MT920447	-
Cophinforma atrovirens	MFLUCC 18-0241	-	-	-	MK340865	-
Curvularia aeria	CBS 294.61	HE861850	-	-	-	HF934812
Curvularia affinis	CBS 154.34	HG778981	-	-	-	HG779159
Curvularia alcornii	MFLUCC 10-0703	NR_137091	-	-	-	-
Curvularia americana	UTHSC:08-3414	NR_146239	-	-	-	HG779200
Curvularia asianensis	MFLUCC 10-0711	JX256424	-	-	-	-
Curvularia australiensis	CBS 126973	MH864363	-	-	-	-
Curvularia australiensis	IMI 53994	KC424595	-	-	-	-
Curvularia beasleyi	BRIP 10972	NR_158442	-	-	-	-
Curvularia beerburrumensis	BRIP 12942	NR_158443	-	-	-	-
Curvularia buchloes	CBS 246.49	NR_147487	-	-	-	-
Curvularia canadensis	CBS 109239	NR_170004	-	-	-	-
Curvularia caricae-papayae	CBS 135941	HG778984	-	-	-	HG779162
Curvularia chiangmaiensis	CPC 28829	MF490814	-	-	-	-

Curvularia chlamydospora	UTHSC 07-2764	HG779021	-	-	-	HG779205
Curvularia chonburiensis	MFLUCC 16-0375	NR_168176	-	-	-	-
Curvularia coatesiae	BRIP 24261	NR_158444	-	-	-	-
Curvularia coicis	CBS 192.29	HF934917	-	-	-	HF934819
Curvularia dactylocteniicola	CPC 28810	MF490815	-	-	-	-
Curvularia eleusinicola	USJCC-0005	MT262877	-	-	-	-
Curvularia ellisii	CBS 193.62	HF934913	-	-	-	HF934815
Curvularia fallax	CBS 155.34	MH855476	-	-	-	-
Curvularia fraserae	BRIP 64708a	NR_185676	-	-	-	-
Curvularia geniculata	CBS 187.50	MH856584	-	-	-	-
Curvularia heteropogonis	CBS 284.91	HF934919	-	-	-	HF934821
Curvularia hominis	UTHSC 09-464	HG779011	-	-	-	HG779191
Curvularia homomorpha	CBS 156.60	MH857938	-	-	-	-
Curvularia ischaemi	CBS 630.82	HG778992	-	-	-	HG779170
Curvularia joliotcurieae	BRIP 14448a	OQ917073	-	-	-	-
Curvularia kenpeggii	BRIP 14530	NR_158447	-	-	-	-
Curvularia khuzestanica	IRAN 3135C	MH688044	-	-	-	-
Curvularia kusanoi	CBS 137.29	NR_165187	-	-	-	LT715733
Curvularia lunata	CBS 730.96	HF934911	-	-	-	HF934813
Curvularia mebaldsii	BRIP 12900	NR_172405	-	-	-	-
Curvularia micrairae	BRIP 17068a	OM421618	-	-	-	-
Curvularia millisiae	BRIP 71718a	OK661031	-	-	-	-
Curvularia miyakei	CBS 197.29	NR_147463	-	-	-	-
Curvularia moringae	CPC 38873	MW175363	-	-	-	MW173117
Curvularia muehlenbeckiae	CBS 144.63	HG779002	-	-	-	HG779180
Curvularia nodosa	CPC 28800	NR_154865	-	-	-	-
Curvularia oryzae	CBS 169.53	HF934906	-	-	-	HF934808
Curvularia ovariicola	CBS 470.90	NR_159856	-	-	-	-
Curvularia patereae	CBS 198.87	NR_169942	-	-	-	-
Curvularia perotidis	CBS 350.90	NR_169942	-	-	-	-
Curvularia pisi	CBS 190.48	NR_152502	-	-	-	-

Curvularia platzii	BRIP27703b	MH414906	-	-	-	-
Curvularia prasadii	CBS 143.64	HG778996	-	-	-	HG779174
Curvularia protuberata	CBS 376.65	HG778998	-	-	-	HG779176
Curvularia pseudobrachyspora	CPC 28808	NR_164423	-	-	-	-
Curvularia pseudoellisii	CBS 298.80	NR_170006	-	-	-	-
Curvularia pseudolunata	UTHSC:09-2092	-	-	-	-	HG779207
Curvularia pseudoprotuberata	CBS 385.69	NR_170008	-	-	-	-
Curvularia robusta	CBS 624.68	HG779000	-	-	-	HG779178
Curvularia ryleyi	CBS 349.90	MH862215	-	-	-	-
Curvularia senegalensis	CBS 149.71	HG779001	-	-	-	HG779179
Curvularia simmonsii	USJCC-0002	MN044753	-	-	-	-
Curvularia soli	CBS 222.96	NR_152503	-	-	-	-
Curvularia sorghina	BRIP 15900	KJ415558	-	-	-	-
Curvularia stenotaphri	BRIP 71303	NR_175746	-	-	-	-
Curvularia subpapendorfii	CBS 656.74	NR_147488	-	-	-	-
Curvularia tanzanica	IMI 507176	NR_173195	-	-	-	-
Curvularia tribuli	CBS 126975	MN688825	-	-	-	-
Curvularia tropicalis	BRIP 14834	KJ415559	-	-	-	-
Curvularia tsudae	ATCC 44764	NR_147464	-	-	-	-
Curvularia tuberculata	CBS 146.63	HF934907	-	-	-	HF934809
Curvularia uncinata	CBS 221.52	HG779024	-	-	-	HG779209
Curvularia variabilis	CPC 28815	NR_154866	-	-	-	-
Curvularia verruciformis	CBS 537.75	HG779026	-	-	-	HG779211
Curvularia warraberensis	BRIP14817	MH414909	-	-	-	-
Diaporthe acaciarum	CBS 138862	KP004460	-	-	-	-
Diaporthe acericola	MFLUCC 17-0956	KY964224	-	KY964137	KY964180	-
Diaporthe actinidiae	ICMP 13683	KC145886	-	-	-	-
Diaporthe alangii	CFCC 52556	MH121491	-	MH121415	MH121533	-
Diaporthe ambigua	CBS 114015	KC343010	-	KC343252	KC343736	-
Diaporthe angelicae	CBS 111592	KC343027	-	KC343269	KC343753	-
Diaporthe arctii	CBS 139280	KJ590736	-	KJ612133	KJ590776	-

Diaporthe arezzoensis	MFLU 19-2880	MT185503	-	-	MT454019	-
Diaporthe arezzoensis	MFLUCC 15-0127	NR_171296	-	-	-	-
Diaporthe australpacifica	BRIP 60163d	OM918688	-	-	OM960597	-
Diaporthe batatas	CBS 122.21	KC343040	-	KC343282	KC343766	-
Diaporthe betulicola	CFCC 51128	KX024653	-	KX024659	KX024655	-
Diaporthe breyniae	CBS 148910	ON400846	-	ON409189	ON409188	-
Diaporthe camporesii	JZB320143	MN535309	-	-	MN984254	-
Diaporthe caryae	CFCC 52563	MH121498	-	MH121422	MH121540	-
Diaporthe caulivora	CBS 127268	KC343045	-	KC343287	KC343771	-
Diaporthe cerradensis = *D. cf. mayteni*	UFMGCB4807	KJ677018	-	-	KP189356	-
Diaporthe cerradensis = *Diaporthe* sp.	CMRP4324	MN173195	-	MW751651	MT311681	-
Diaporthe cerradensis = *Diaporthe* sp.	CMRP4331	MN173198	-	MW751655	MT311685	-
Diaporthe cerradensis= *Diaporthe* sp.	LGMF1616	MG976419	-	-	-	-
Diaporthe chinensis	MFLUCC 19-0101	MW187324	-	MW294199	MW205017	-
Diaporthe cucurbitae	DAOM 42078	KM453210	-	-	KM453211	-
Diaporthe cuppatea	CBS 117499	KC343057	-	KC343299	KC343783	-
Diaporthe drenthii	BRIP 66524	MN708229	-	-	MN696526	-
Diaporthe endophytica	CBS 133811	KC343065	-	KC343307	KC343791	-
Diaporthe etinsideae	BRIP 64096a	OM918692	-	-	OM960601	-
Diaporthe fici-septicae	MFLU 18-2588	MW114348	-	-	MW192211	-
Diaporthe goulteri	BRIP 55657a	KJ197290	-	-	KJ197252	-
Diaporthe griceae	BRIP 67014a	OM918694	-	-	OM960603	-
Diaporthe guangdongensis	ZHKUCC20-0014	MT355684	-	MT409314	MT409338	-
Diaporthe gulyae	BRIP 54025	JF431299	-	-	JN645803	-
Diaporthe hubeiensis	JZB320123	MK335809	-	MK500235	MK523570	-
Diaporthe infecunda	CBS 133812	KC343126	-	KC343368	KC343852	-
Diaporthe kongii	BRIP 54031	JF431301	-	-	JN645797	-
Diaporthe longicolla	ATCC 60325	KJ590728	-	KJ612124	KJ590767	-
Diaporthe longispora	CBS 194.36	KC343135	-	KC343377	KC343861	-
Diaporthe lusitanicae	CBS 123212	KC343136	-	KC343378	KC343862	-
Diaporthe lusitanicae	CBS 123213	KC343137	-	KC343379	KC343863	-

Diaporthe machili	SAUCC 194.111	-	-	-	-	-
Diaporthe malorum	CAA734	KY435638	-	KY435658	KY435627	-
Diaporthe masirevicii	BRIP 57892a	KJ197277	-	-	KJ197239	-
Diaporthe mayteni	CBS 133185	KC343139	-	KC343139	KC343865	-
Diaporthe melonis	CBS 507.78	KC343141	-	KC343383	KC343867	-
Diaporthe miriciae	BRIP 54736j	KJ197282	-	-	KJ197244	-
Diaporthe monetii	MF-Ha18-049	MW008494	-	MZ671939	MW008516	-
Diaporthe myracrodruonis	URM7972	MK205289	-	MK205290	MK213408	-
Diaporthe neoarctii	CBS 109490	KC343145	-	KC343387	KC343871	-
Diaporthe novem	CBS 127270	KC343156	-	KC343398	KC343882	-
Diaporthe novem	CBS 127271	KR061990	-	KC343399	KC343883	-
Diaporthe oxe	CBS 133186	KC343164	-	KC343406	KC343890	-
Diaporthe phaseolorum	AR4203	KJ590738	-	KJ612135	KJ590739	-
Diaporthe pyracanthae	CAA483	KY435635	-	KY435656	KY435625	-
Diaporthe racemosae	CPC 26646	MG600223	-	MG600219	MG600225	-
Diaporthe raonikayaporum	CBS 133182	KC343188	-	KC343430	KC343914	-
Diaporthe rosae	MFLUCC 17-2658	MG828894	-	MG829273	-	-
Diaporthe sackstonii	BRIP 54669b	KJ197287	-	-	KJ197249	-
Diaporthe sapindicola	CFCC 55344	MW881507	-	MW898943	MW898934	-
Diaporthe schini	CBS 133181	KC343191	-	KC343433	KC343917	-
Diaporthe sclerotioides	CBS 296.67	KC343193	-	KC343435	KC343919	-
Diaporthe searlei	BRIP 66528	MN708231	-	-	-	-
Diaporthe sojae	CBS 139282	NR_147542	-	-	-	-
Diaporthe stewartii	CBS 193.36	FJ889448	-	JX197415	GQ250324	-
Diaporthe tectonae	MFLUCC 12-0777	KU712430	-	KU749345	KU749359	-
Diaporthe terebinthifolii	CBS 133180	KC343216	-	KC343458	KC343942	-
Diaporthe thunbergiicola	MFLUCC 12-0033	KP715097	-	-	KP715098	-
Diaporthe trevorrowii	BRIP 70737a	OM918703	-	-	OM960612	-
Diaporthe tulliensis	BRIP 62248a	KR936130	-	-	KR936133	-
Diaporthe ueckeri	CBS 139283	NR_147543	-	-	-	-
Diaporthe vangoghii	MF-Ha18-046	MW008492	-	MZ671937	MW008514	-

Diaporthe yunnanensis	CGMCC 3.18289	KX986796	-	KX999290	KX999188	-
Annulohypoxylon truncatum	EKTX14006	KX376329	KX376352	-	-	-
Hypomontagnella barbarensis	STMA 14081	MK131720	MK135893	-	-	-
Hypoxylon erythrostroma	MUCL 53759	KC968910	KC977296	-	-	-
Hypoxylon addis	MUCL 52797	KC968931	KC977287	-	-	-
Hypoxylon anthochroum	YBY-F117	OP704210	-	-	-	-
Hypoxylon aveirense	CMG 29	MN053021	MN066636	-	-	-
Hypoxylon aveirense	MUM 19.40	NR_173851	-	-	-	-
Hypoxylon baruense	UCH9545	MN056428	MK908142	-	-	-
Hypoxylon begae	Voucher 215(JDR)	JN660820	AY951704	-	-	-
Hypoxylon bellicolor	UCH9543	MN056425	MK908139	-	-	-
Hypoxylon brevisporum	Voucher 36 (JDR)	JN660821	AY951705	-	-	-
Hypoxylon calileguense	STMA 14059	KU604566	KU604579	-	-	-
Hypoxylon carneum	MUCL 54177	KY610400	KX271270	-	-	-
Hypoxylon cercidicola	CBS 119009	KU683766	KU684189	-	-	-
Hypoxylon chrysalidosporum	FCATAS 2710	OL467295	OL584230	-	-	-
Hypoxylon cinnabarinum	UCH9546	MN056429	MK908143	-	-	-
Hypoxylon damuense	FCATAS 4207	ON075427	ON093245	-	-	-
Hypoxylon dieckmannii	YMJ 89041203	JN979413	AY951713	-	-	-
Hypoxylon eurasiaticum	MUCL 57720	NR_172358	-	-	-	-
Hypoxylon fendleri	DSM:107927	MK287533	MK287571	-	-	-
Hypoxylon fendleri	UCH9552	MN056423	MK908137	-	-	-
Hypoxylon ferrugineum	ZT-Myc-64240	MW489546	-	-	-	-
Hypoxylon flavoargillaceum	STMA 14062	KU604577	KU159532	-	-	-
Hypoxylon fragiforme	MUCL 51264	KC477229	KX271282	-	-	-
Hypoxylon fraxinophilum	MUCL 54176	KC968938	KC977301	-	-	-
Hypoxylon fulvosulphureum	EK13010	KP401576	KP401584	-	-	-
Hypoxylon fuscum	CBS 113049	NR_172215	-	-	-	-
Hypoxylon gibriacense	MUCL 52698	JX658523	ON813074	-	-	-
Hypoxylon griseobrunneum	CBS 331.73	KY610402	KC977303	-	-	-
Hypoxylon haematostroma	MUCL 47600	AM749924	KC977279	-	-	-

Hypoxylon haematostroma	UCH9555	MN056424	MK908138	-	-	-
Hypoxylon hinnuleum	CBS 286.62	MH858152	-	-	-	-
Hypoxylon hinnuleum	MUCL:3621	MK287537	MK287575	-	-	-
Hypoxylon howeanum	UCH9565	MN056427	MK908141	-	-	-
Hypoxylon hypomiltum	MUCL 53312	KC968914	KC977298	-	-	-
Hypoxylon investiens	CBS 118183	FJ185307	FJ185298	-	-	-
Hypoxylon investiens	CBS 118185	FJ185308	FJ185299	-	-	-
Hypoxylon investiens	MUCL 53307	KC477239	KC977293	-	-	-
Hypoxylon isabellinum	MUCL 53308	KC968935	KC977295	-	-	-
Hypoxylon jaklitschii	CBS 138916	NR_158468	-	-	-	-
Hypoxylon jaklitschii	JF13037	KM610290	KM610304	-	-	-
Hypoxylon jecorinum	YMJ39	JN979429	AY951731	-	-	-
Hypoxylon lateripigmentum	MUCL 53304	NG_059786	-	-	-	-
Hypoxylon lignicola	MFLUCC 16-0926	MK828609	-	-	-	-
Hypoxylon lilloi	STMA 14142	KU604574	KU159537	-	-	-
Hypoxylon liviae	CBS 115282	KC968922	KC977265	-	-	-
Hypoxylon lividicolor	YMJ 70	JN979432	AY951734	-	-	-
Hypoxylon lividipigmentum	YMJ 233	JN979433	AY951735	-	-	-
Hypoxylon macrosporum	YMJ 47	JN979434	AY951736	-	-	-
Hypoxylon mangrovei	MFLU 18-0559	MN047116	MN077053	-	-	-
Hypoxylon medogense	FCATAS4061	ON075425	ON093243	-	-	-
Hypoxylon munkii	MUCL:53315	KC968912	KC977294	-	-	-
Hypoxylon musceum	MUCL:53765	KC968926	KC977280	-	-	-
Hypoxylon neosublenormandii	MFLU 15-1193	NR_155174	-	-	-	-
Hypoxylon neosublenormandii	MFLUCC 11-0618	KU940157	-	-	-	-
Hypoxylon ochraceum	MUCL:54625	KC968937	KC977300	-	-	-
Hypoxylon olivaceopigmentum	DSM:107924	MK287530	MK287568	-	-	-
Hypoxylon papillatum	ATCC:58729	KC968919	KC977258	-	-	-
Hypoxylon perforatum	MUCL 54174	KC968936	KC977299	-	-	-
Hypoxylon petriniae	CBS 114746	KY610405	KX271274	-	-	-
Hypoxylon pilgerianum	YMJ 92042505	JQ009310	AY951744	-	-	-

Hypoxylon polyporoideum	YMJ 15	JQ009311	AY951747	-	-	-
Hypoxylon porphyreum	CBS:119022	KC968921	KC977264	-	-	-
Hypoxylon pseudofuscum	DSM 112038	MW367857	MW373867	-	-	-
Hypoxylon pulicicidum	CBS 122622	JX183076	JX183074	-	-	-
Hypoxylon pulicicidum	MUCL49879	JX183075	JX183072	-	-	-
Hypoxylon rickii	MUCL:53309	KY610416	KC977288	-	-	-
Hypoxylon rubiginosum	MUCL 52887	KC477232	KY624311	-	-	-
Hypoxylon samuelsii	MUCL:51843	KC968916	KC977286	-	-	-
Hypoxylon spegazzinianum	STMA 14082	KU604573	KU604582	-	-	-
Hypoxylon sporistriatatunicum	UCH9542	MN056426	MK908140	-	-	-
Hypoxylon subgilvum	YMJ 246	JQ009314	AY951754	-	-	-
Hypoxylon subrutiloides	F202416	FJ185304	FJ185281	-	-	-
Hypoxylon ticinense	YMJ 313	JQ009317	AY951757	-	-	-
Hypoxylon trugodes	MUCL 54794	KF234422	KF300548	-	-	-
Hypoxylon ulmophilum	YMJ 350	JQ009320	AY951760	-	-	-
Hypoxylon vogesiacum	CBS 115273	KY610417	KX271275	-	-	-
Hypoxylon wuzhishanense	FCATAS2708	OL467292	OL584227	-	-	-
Jackrogersella multiformis	CBS 119016	KC477234	KX271262	-	-	-
Neofusicoccum dianense	CSF6075	MT028605	MT028937	-	MT028771	-
Neofusicoccum hellenicum	CERC 1947	KP217053	KP217069	-	KP217061	-
Neofusicoccum kwambonambiense	CBS 123639	MH863317	-	-	-	-
Neofusicoccum kwambonambiense	CMW14023	EU821900	EU821840	-	EU821870	-
Neofusicoccum magniconidium	CGMCC 3.20077	NR_172859	-	-	-	-
Neofusicoccum magniconidium	CSF5876	MT028612	MT028944	-	MT028778.	-
Neofusicoccum ningerense	CGMCC 3.20078	NR_172860	-	-	-	-
Neofusicoccum nonquaesitum	PD484	-	GU251823	-	-	-
Neofusicoccum nonquaesitum	CBS 126655	MH864187	-	-	-	-
Neofusicoccum occulatum	CBS 128008	MH864743	EU339472	-	EU339509	-
Neofusicoccum podocarpi	CBS 131677	MT587508	MT592715	-	MT592223	-
Neofusicoccum sinoeucalypti	CERC 2005	KX278061	KX278270	-	KX278166	-
Neofusicoccum vitifusiforme	CBS 110887	MH862869	KX465061	-		-

Neofusicoccum yunnanense	CSF6142	MT028667	MT028999	-	MT028833	-
Neoscytalidium dimidiatum	CBS 499.66	KF531820	KF531800	-	-	-
Neoscytalidium dimidiatum	CBS 125608	-	MT592752	-	MT592260	-
Neoscytalidium hyalinum	CBS 145.78	MH872880	-	-	-	-
Neoscytalidium hyalinum	CBS 125807	MH863770	-	-	-	-
Neoscytalidium hyalinum	IRNHM-KZN2	MG220374	-	-	MG220381	-
Neoscytalidium hyalinum	IRNHM-KZN4	MG220376	-	-	MG220383	-
Neoscytalidium hylocereum	TSU-HP01	-	LC647833	-	-	-
Neoscytalidium hylocereum	PSU-HP01	-	LC647832	-	-	-
Neoscytalidium novaehollandiae	CBS 122071	MH863173	MT592760	-	-	-
Neoscytalidium novaehollandiae	WAC 12691	NR_111260	-	-	-	-
Neoscytalidium oculi	MX89	JQ905748	-	-	-	-
Neoscytalidium orchidacearum	MFLUCC 12-0533	KU179865	-	-	-	-
Pyrenopolyporus laminosus	MUCL 53305	KC968934	KC977292	-	-	-
Rostrohypoxylon terebratum	CBS 119137	DQ631943	DQ840097	-	-	-
Penicillium aethiopicum	CBS 484.84	-	JF909958	-	-	JF909940
Penicillium allii-sativi	DTO 149A8	-	JX996891	-	-	JX996627
Penicillium carneum	CBS 112297	-	AY674386	-	-	JN406642
Penicillium chrysogenum	CBS 306.48	-	AY495981	-	-	JF909937
Penicillium clavigerum	CBS 25594	-	AY674427	-	-	-
Penicillium coccotrypicola	BRIP 59608	-	KM605437	-	-	-
Penicillium confertum	CBS 171.87	-	JF909952	-	-	JF909934
Penicillium desertorum	DTO 148I6	-	JX996818	-	-	JX996682
Penicillium digitatum	CBS 112082	-	KJ834447	-	-	JN121426
Penicillium dipodomyis	CBS 110412	-	JF909950	-	-	JX996695
Penicillium egyptiacum	CBS 244.32	-	JX996845	-	-	JX996714
Penicillium expansum	CBS 32548	-	AY674400	-	-	-
Penicillium flavigenum	CBS 419.89	-	JF909957	-	-	JF909939
Penicillium glycyrrhizacola	G4432	-	KF021538	-	-	KF021554
Penicillium goetzii	CBS 285.73	-	JX996847	-	-	JX996716
Penicillium halotolerans	DTO 148H9	-	JX996816	-	-	JX996680

Penicillium harmonense	CBS 412.69	-	AY495996	-	-	-
Penicillium italicum	CBS 33948	-	AY674398	-	-	-
Penicillium kewense	CBS 344.61	-	JX996849	-	-	JX996718
Penicillium lanosocoeruleum	CBS 215.30	-	JX996843	-	-	JX996712
Penicillium marinum	CBS 109550	-	AY674392	-	-	KU904357
Penicillium mediterraneum	FMR 15188	-	LT898291	-	-	LT899802
Penicillium mononematosum	CBS 172.87	-	JF909953	-	-	JF909935
Penicillium nalgiovense	CBS 352.48	-	JX996850	-	-	JX996719
Penicillium nalgiovense	NRRL 911	-	AY371601	-	-	-
Penicillium osmophilum	CBS 462.72	-	MN969391	-	-	JN121518
Penicillium paneum	CBS 101032	-	AY674387	-	-	KU904361
Penicillium persicinum	CBS 111235	-	JF909951	-	-	JF909933
Penicillium psychrosexualis	CBS 128137	-	HQ442356	-	-	KU904362
Penicillium roqueforti	CBS 221.30	-	MN969396	-	-	JN406611
Penicillium rubens	DTO 98E8	-	JF909949	-	-	JF909931
Penicillium samsonianum	AS3.15403	-	KJ668582	-	-	KT698899
Penicillium sclerotigenum	CBS 101033	-	AY674393	-	-	JN406652
Penicillium sinaicum	CBS 279.82	-	JX996846	-	-	JX996715
Penicillium tardochrysogenum	DTO 149B9	-	JX996898	-	-	JX996634
Penicillium ulaiense	CBS 21092	-	AY674408	-	-	-
Penicillium xingjiangense	CGMCC 3.15274	-	KF021544	-	-	KF021557

[1]GenBank - Internal transcribed spacer regions (ITS), β-tubulin (*tub2*), calmodulin (*cal*), partial elongation factor 1-alpha gene (*tef1*), and the second largest protein subunit of DNA-directed RNA polymerase II (*rpb2*).

-: Sequence not available.

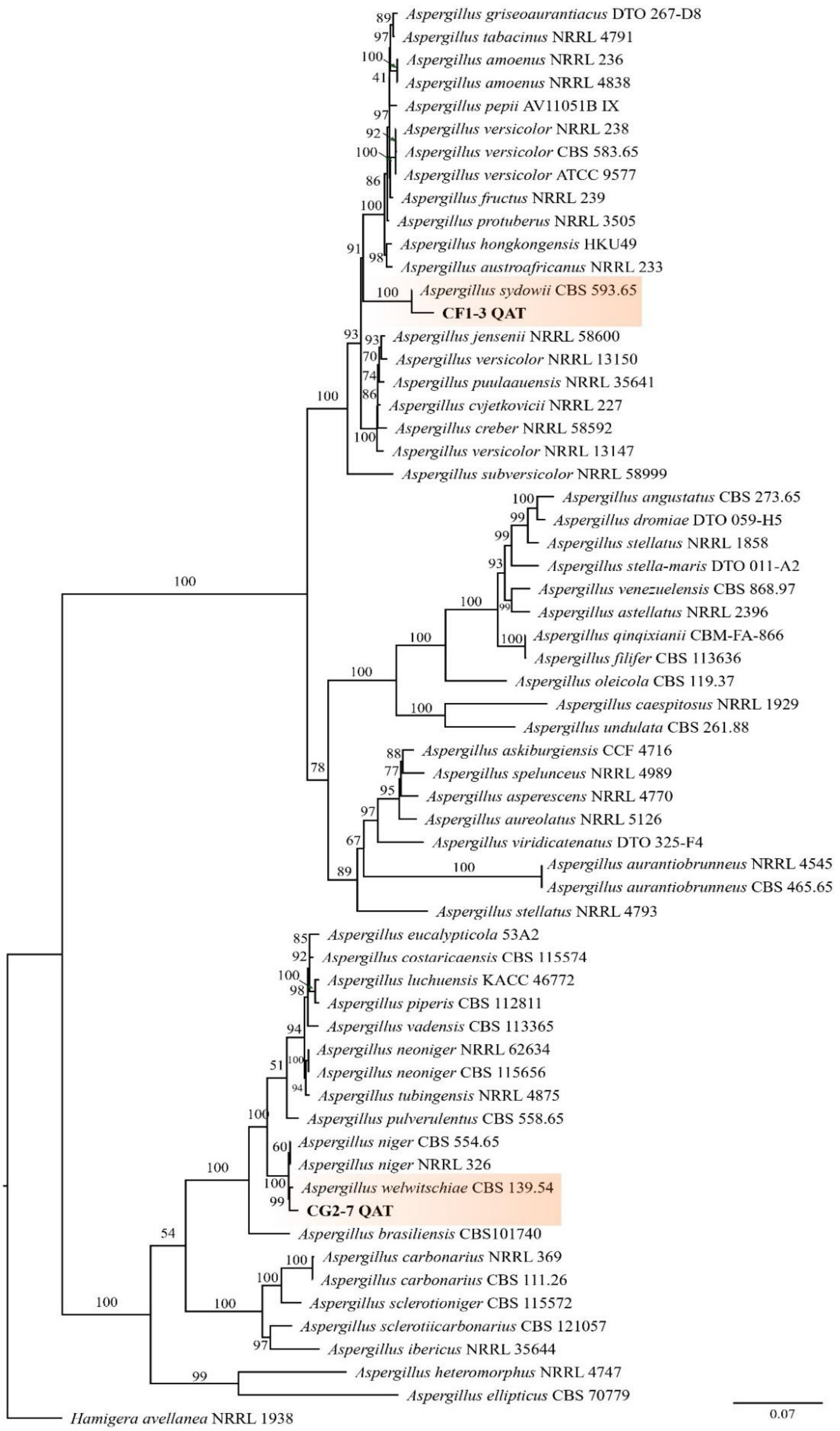

Figure S1. Concatenated phylogenetic analysis by Maximum Likelihood (ML) of the endophytic fungi CF1-3, isolated from *Fridericia chica* leaves, and CG2-7, isolated from *F. chica* branches, based on partial sequences of ITS, *tub*2, *cal* and *rpb*2. The scale bar of 0.07 represents the number of changes and the number indicates the bootstrap support of the branches. The sequences of the isolates studied here are highlighted in bold. The tree was rooted in *Hamigera avellanea* NRRL 1938.

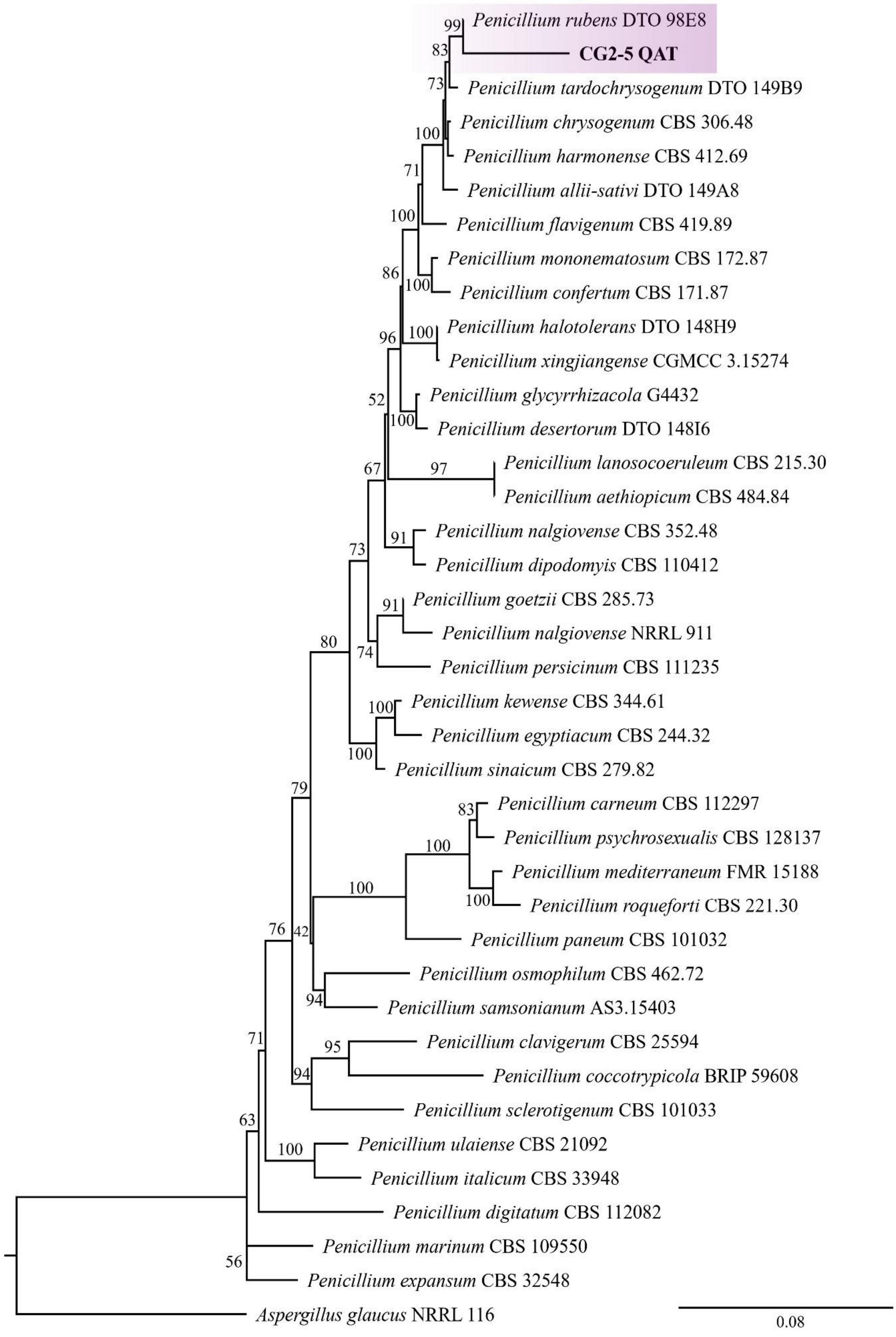

Figure S2. Concatenated phylogenetic analysis by Maximum Likelihood (ML) of the endophytic fungus CG2-5, isolated from *Fridericia chica* branches, based on partial sequences of *tub*2 and *rpb*2. The scale bar of 0.08 represents the number of changes and the number indicates the bootstrap support of the branches. The sequences of the isolates studied here are highlighted in bold. The tree was rooted in A*spergillus glaucus* NRRL 116.

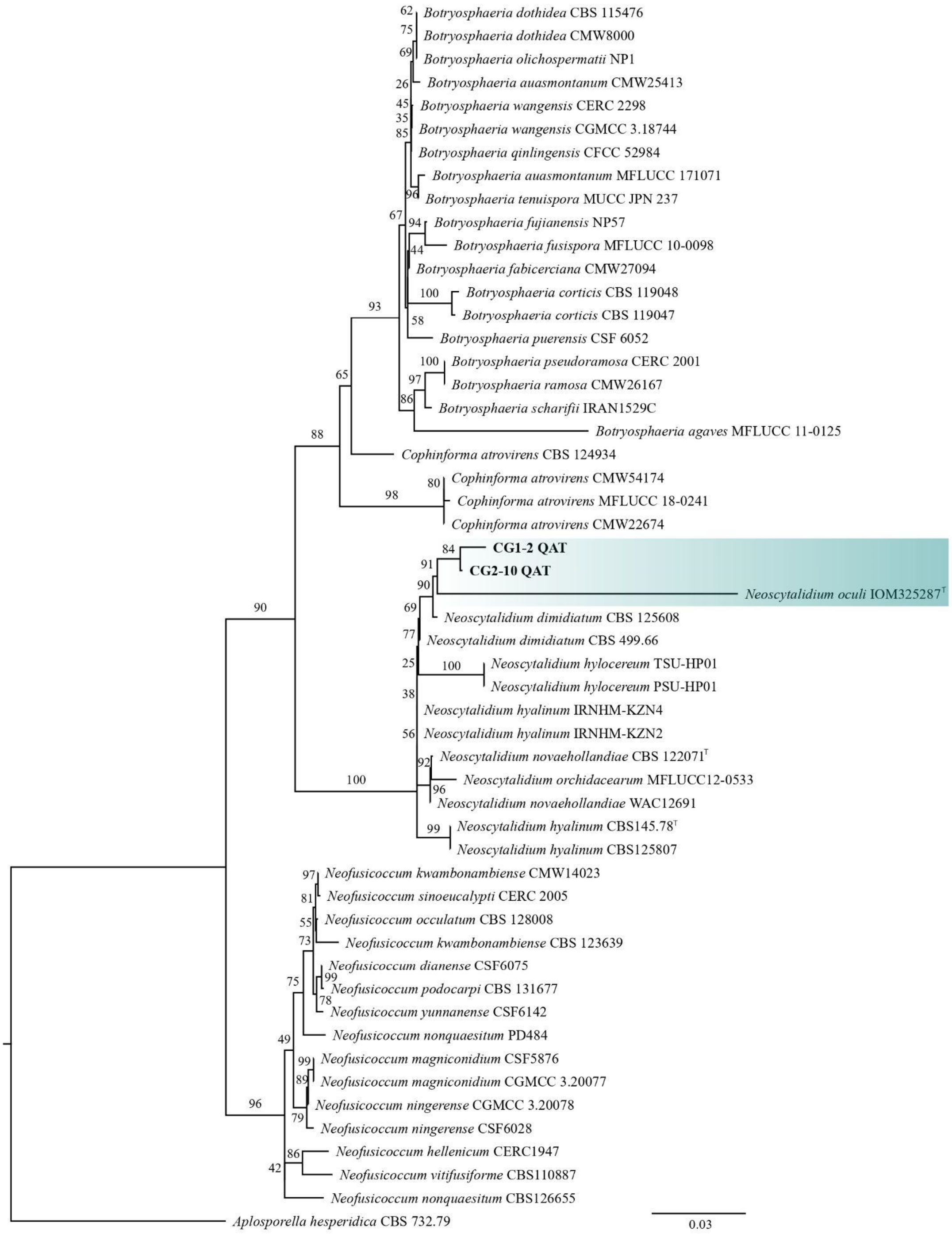

Figure S3. Concatenated phylogenetic analysis by Maximum Likelihood (ML) of the endophytic fungi CG1-2 and CG2-10, both isolated from branches of *Fridericia chica*, based on partial sequences of ITS, *tub*2 and *tef*1. The scale bar of 0.03 represents the number of changes and the number indicates the bootstrap support of the branches. The sequences of the isolates studied here are highlighted in bold. The tree was rooted in *Aplosporella hesperidica* CBS 732.79.

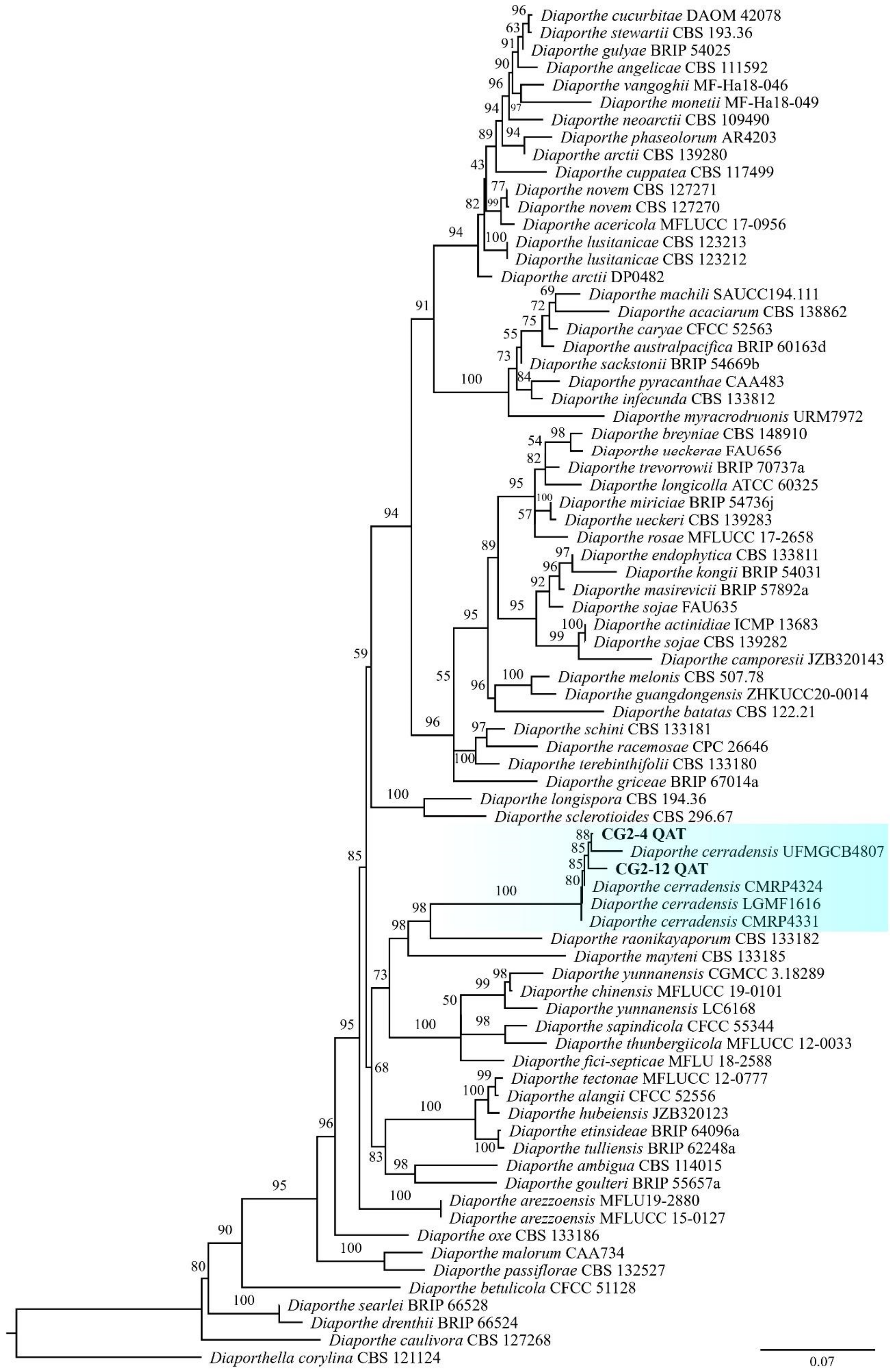

Figure S4. Concatenated phylogenetic analysis by Maximum Likelihood (ML) of the endophytic fungi CG2-4 and CG2-12, both isolated from branches of *Fridericia chica*, based on partial sequences of ITS, *cal* and *tef*1. The scale bar of 0.07 represents the number of changes and the number indicates the bootstrap support of the branches. The sequences of the isolates studied here are highlighted in bold. The tree was rooted in *Diaporthella corylina* CBS 121124.

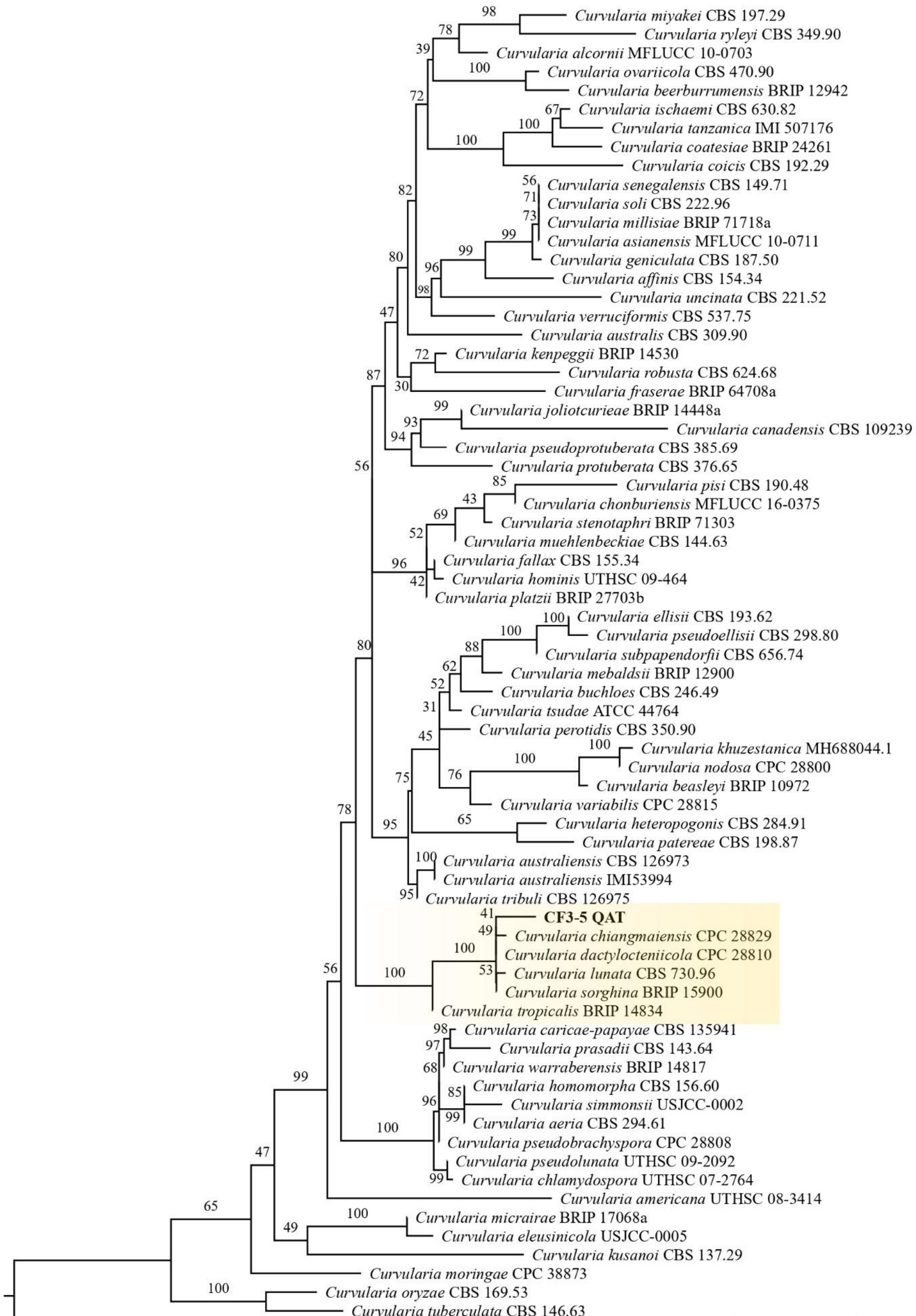

Figure S5. Concatenated phylogenetic analysis by Maximum Likelihood (ML) of the endophytic fungus CF3-5, isolated from the leaves of *Fridericia chica*, based on partial sequences of ITS and *rpb2*. The scale bar of 0.03 represents the number of changes and the number indicates the bootstrap support of the branches. The sequences of the isolates studied here are highlighted in bold. The tree was rooted in *Alternaria alternata* CBS 916.96.

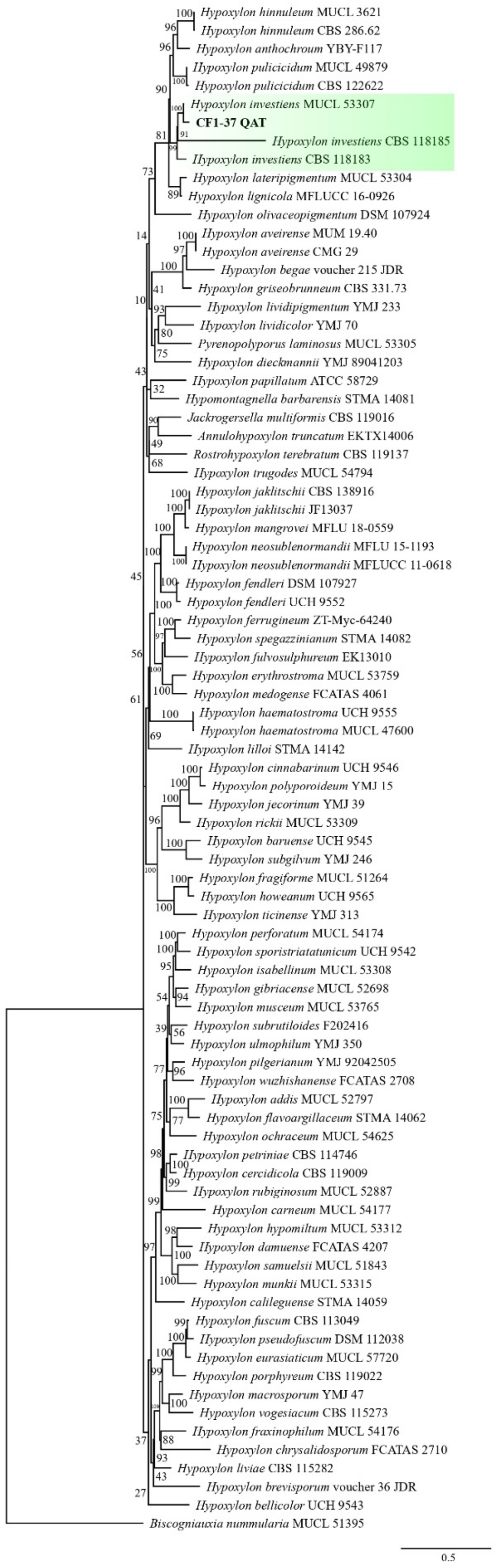

Figure S6. Concatenated phylogenetic analysis by Maximum Likelihood (ML) of the endophytic fungus CF1-37, isolated from the leaves of *Fridericia chica*, based on partial sequences of ITS and *tub2*. The scale bar of 0.5 represents the number of changes and the number indicates the bootstrap support of the branches. The sequences of the isolates studied here are highlighted in bold. The tree was rooted in *Biscogniauxia nummularia* MUCL 51395.
